# Neuronal DAMPs exacerbate neurodegeneration via astrocytic RIPK3 signaling

**DOI:** 10.1172/jci.insight.177002

**Published:** 2024-05-07

**Authors:** Nydia P. Chang, Evan M. DaPrano, Marissa Lindman, Irving Estevez, Tsui-Wen Chou, Wesley R. Evans, Marialaina Nissenbaum, Micheal McCourt, Diego Alzate, Colm Atkins, Alexander W. Kusnecov, Rafiq Huda, Brian P. Daniels

**Affiliations:** 1 Department of Cell Biology and Neuroscience,; 2 W. M. Keck Center for Collaborative Neuroscience, and; 3 Department of Psychology, Rutgers University, Piscataway, New Jersey, USA.

**Keywords:** Immunology, Neuroscience, Innate immunity, Neurodegeneration

## Abstract

Astrocyte activation is a common feature of neurodegenerative diseases. However, the ways in which dying neurons influence the activity of astrocytes is poorly understood. Receptor interacting protein kinase-3 (RIPK3) signaling has recently been described as a key regulator of neuroinflammation, but whether this kinase mediates astrocytic responsiveness to neuronal death has not yet been studied. Here, we used the 1-methyl-4-phenyl-1, 2, 3, 6-tetrahydropyridine model of Parkinson’s disease to show that activation of astrocytic RIPK3 drives dopaminergic cell death and axon damage. Transcriptomic profiling revealed that astrocytic RIPK3 promoted gene expression associated with neuroinflammation and movement disorders, and this coincided with significant engagement of damage-associated molecular pattern signaling. In mechanistic experiments, we showed that factors released from dying neurons signaled through receptor for advanced glycation endproducts to induce astrocytic RIPK3 signaling, which conferred inflammatory and neurotoxic functional activity. These findings highlight a mechanism of neuron-glia crosstalk in which neuronal death perpetuates further neurodegeneration by engaging inflammatory astrocyte activation via RIPK3.

## Abstract 2

Factors released from dying neurons activate necroptosis-independent RIPK3 signaling in astrocytes, driving neurotoxic activation and exacerbating neurodegeneration.

## Introduction

Recent work has identified a central role for neuroinflammation in the pathogenesis of neurological disease, including major neurodegenerative disorders such as Alzheimer’s and Parkinson’s disease (1, 2). Although glial cells are critical regulators of neuroinflammation, activated glia serve complex roles during disease, including both protective and pathogenic functions (3). Among glial cells, astrocytes are the most abundant cell type in the central nervous system (CNS), where they support homeostasis via wide-ranging effects on neurotransmission, neurovascular function, and metabolism (4). However, following an inflammatory insult, astrocytes can enter “reactive” states associated with disease pathogenesis (5). Although astrocyte activation is likely highly plastic and context dependent, it is now widely accepted that astrocytes can take on inflammatory transcriptional states during disease that are associated with the conferral of neurotoxic activity and suppression of normal homeostatic functions (6). Despite this understanding, the molecular mechanisms that govern astrocyte reactivity during neurodegenerative disease, and particularly those factors that most directly exacerbate disease progression, remain poorly understood (7).

We and others have recently identified receptor interacting protein kinase-3 (RIPK3) as a key regulator of inflammation in the CNS (8–10). RIPK3 signaling is canonically associated with necroptotic cell death, which is induced via the activation of mixed lineage kinase domain-like protein (MLKL) (11). Although RIPK3-dependent necroptosis has been implicated in several neurological disorders, RIPK3 also appears to promote neuroinflammatory processes via necroptosis-independent mechanisms, including the coordination of inflammatory transcription in multiple CNS cell types (12–17). Although necroptosis-independent roles for RIPK3 signaling in astrocytes have not been thoroughly studied, we have previously shown that pathogenic α-synuclein fibrils activate RIPK3 signaling in human midbrain astrocyte cultures, resulting in NF-κB–mediated transcriptional activation without inducing astrocytic necroptosis (14). However, whether RIPK3 controls astrocyte transcriptional activation and function in models of neurodegenerative disease in vivo is unknown.

The importance of neuron-glia communication during CNS disease states has also gained significant recognition in recent work (18). A particularly important goal in this area is defining the stimuli that induce inflammatory signaling in the “sterile” setting of neurodegeneration. Potential stimuli underlying inflammatory astrocyte activation during neurodegeneration are factors derived from dead and dying neurons themselves. These factors include damage-associated molecular patterns (DAMPs), molecules released from damaged cells that serve as endogenous danger signals that elicit potent innate immune activation in neighboring cells (19). DAMP release has been associated with numerous inflammatory diseases, including neurodegenerative disorders (20–23). However, whether and how neuron-derived DAMPs affect astrocyte function during neurodegenerative disease have not been thoroughly studied to date.

Here, we define a role for RIPK3 signaling in mediating astrocyte activation downstream of neuronal DAMP release. We utilize the 1-methyl-4-phenyl-1, 2, 3, 6-tetrahydropyridine (MPTP) model of Parkinson’s disease, in which cell death can be selectively induced in dopaminergic neurons in vivo, to show that induction of neuronal cell death results in RIPK3-dependent astrocyte activation, which in turn exacerbates ongoing neurodegeneration. Transcriptional profiling revealed a robust RIPK3-dependent inflammatory signature in astrocytes exposed to dying neuron–derived factors, and this occurred independently of astrocytic MLKL. Mechanistically, we showed that factors released from dying dopaminergic neurons activated receptor for advanced glycation endproducts (RAGE) on midbrain astrocytes. RAGE signaling, in turn, was required for RIPK3 activation, inflammatory transcription, and the conferral of neurotoxic activity in midbrain astrocytes following exposure to neuronal DAMPs. Our findings suggest a feed-forward mechanism that perpetuates neurodegeneration via the DAMP-dependent activation of RIPK3-dependent inflammation and neurotoxicity in astrocytes. These results highlight an important mechanism of neuron-glia crosstalk, with implications for the prevention and treatment of neurodegenerative disease.

## Results

### Astrocytic RIPK3 signaling promotes neurodegeneration in the MPTP model of Parkinson’s disease.

**Figure 1:**
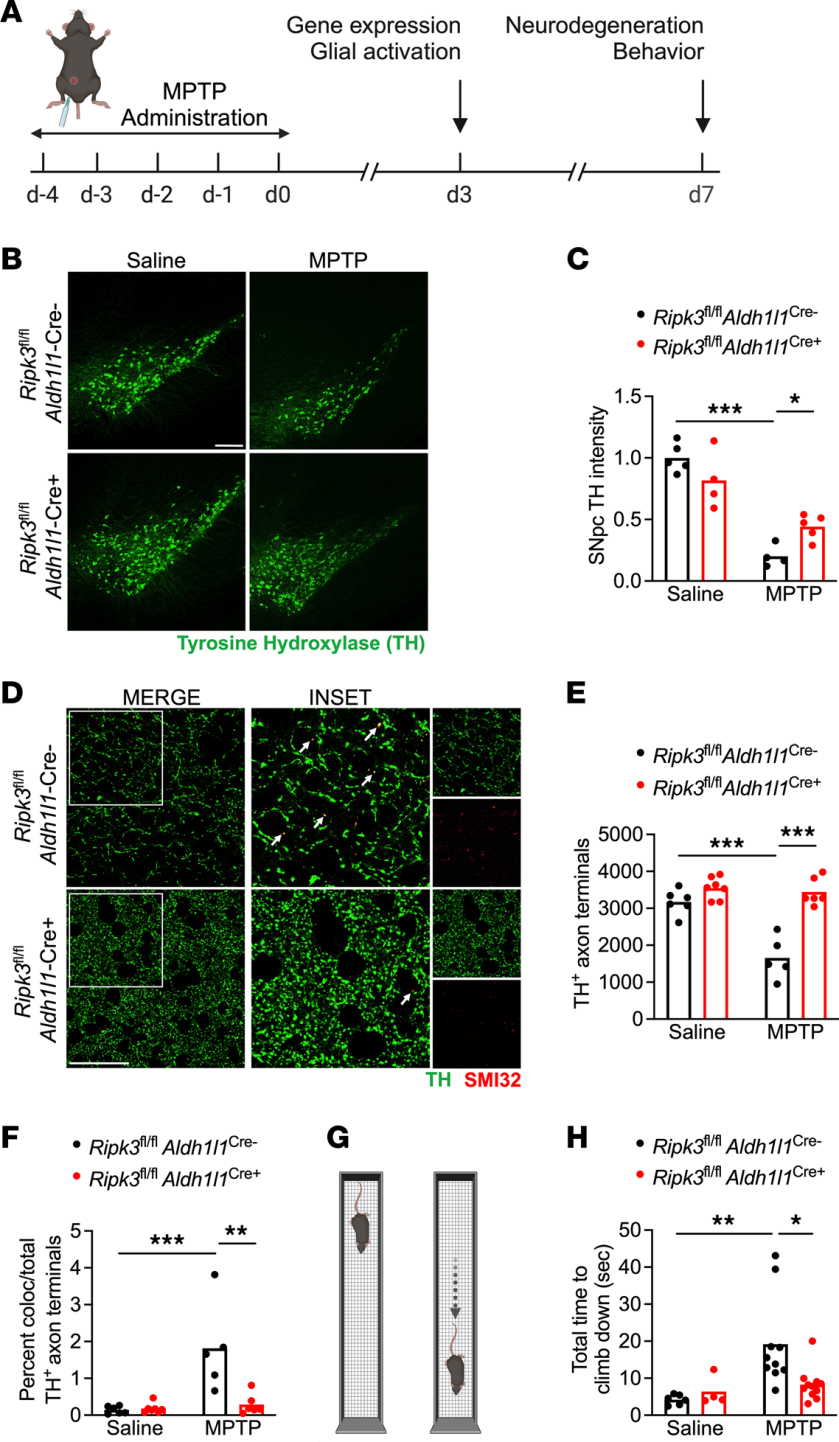
Astrocytic RIPK3 signaling promotes neurodegeneration in the MPTP model of Parkinson’s disease. (**A**) Schematic diagram showing treatment paradigm for the subacute MPTP model with selected experimental endpoints used in this study. (**B** and **C**) IHC analysis of tyrosine hydroxylase (TH) staining in the substantia nigra pars compacta (SNpc) in indicated genotypes 7 days following either saline or MPTP treatment (scale bar = 200 μm). (**D**–**F**) IHC analysis of TH^+^ axons with colabeling with the damaged axon marker SMI32 in the striatum in indicated genotypes 7 days following either saline or MPTP treatment (scale bar = 20 μm). Insets represent 2× digital zoom of the original 40× images. Arrows represent colocalized puncta for both TH and SMI32 staining. (**G**) Schematic diagram for the vertical grid test. (**H**) Behavioral performance in the vertical grid test 7 days after injection with MPTP or saline. *n* = 4–5 mice/group (**B** and **C**), 5–7 mice/group (**D**–**F**), 4–11 mice/group (**H**). Data are represented as mean values with scatterplots depicting individual biological replicate values. All comparisons via 2-way ANOVA with Holm-Šídák multiple-comparison test. *P < 0.05, **P < 0.01, ***P < 0.001. **A** and **G** were created with Biorender.com.

To examine the impact of astrocytic RIPK3 signaling in response to neuronal cell death, we subjected mice with astrocyte-specific deletion of *Ripk3* (*Ripk3*^fl/fl^ *Aldh1l1*^Cre+^) and littermate controls to treatment with MPTP, a neurotoxin that selectively induces death in dopaminergic neurons (24, 25). We used the subacute model of MPTP administration, in which mice receive 5 daily doses at 20 mg/kg intraperitoneally (i.p.), followed by downstream analysis as depicted in Figure 1A. MPTP administration resulted in significant loss of tyrosine hydroxylase (TH) immunoreactivity in the substantia nigra pars compacta (SNpc) of control animals, consistent with the depletion of dopaminergic neurons in this region (Figure 1, B and C). Strikingly, however, *Ripk3*^fl/fl^ *Aldh1l1*^Cre+^ mice exhibited reduced dopaminergic neuron loss following MPTP treatment, suggesting a role for astrocytic RIPK3 in exacerbating neuronal death in this model. We also observed a significant loss of TH^+^ dopaminergic axons in the striatum of control animals (Figure 1, D and E), along with increased frequencies of TH^+^ axons immunoreactive for SMI32, a marker of axonal degeneration (26–28) (Figure 1F). This phenotype was also greatly ameliorated in *Ripk3*^fl/fl^ *Aldh1l1*^Cre+^ mice. To test whether these differences in dopaminergic neuron loss were associated with differences in motor function, we next subjected mice to the vertical grid maze, a motor task previously shown to be sensitive to perturbations of dopaminergic circuits (29, 30). Strikingly, MPTP-treated control mice exhibited significantly impaired performance in the vertical grid maze (Figure 1, G and H), while mice lacking astrocytic *Ripk3* did not. Improvements in dopaminergic neuron loss and motor performance in *Ripk3*^fl/fl^ *Aldh1l1*^Cre+^ mice were not due to differential metabolism of MPTP compared to Cre^–^ littermates, as we observed indistinguishable levels of the toxic metabolite of MPTP (MPP^+^) in midbrain homogenates derived from animals of both genotypes (Supplemental Figure 1A; supplemental material available online with this article; https://doi.org/10.1172/jci.insight.177002DS1). We also verified that *Ripk3* transcript expression was absent in sorted astrocyte cell surface antigen-2–positive (ACSA2^+^) astrocytes derived from *Ripk3*^fl/fl^ *Aldh1l1*^Cre+^ mice, while *Ripk3* expression in sorted CD11b^+^ cells was unchanged (Supplemental Figure 1, B–D). Together, these data suggest that astrocytic RIPK3 signaling exacerbates neuronal cell death following a neurotoxic insult.

### RIPK3 drives inflammatory transcriptional activation but not proliferation in midbrain astrocytes.

**Figure 2:**
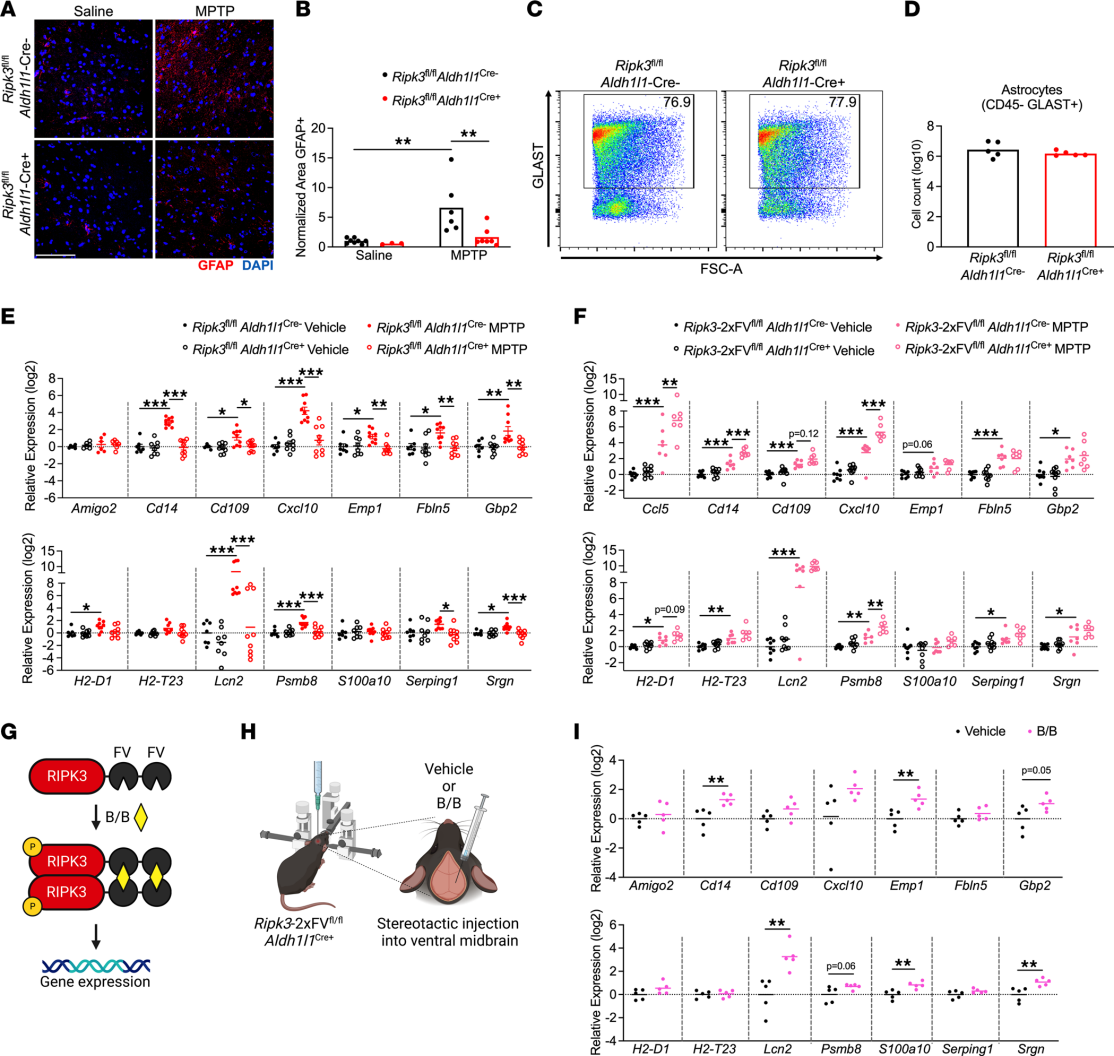
RIPK3 drives inflammatory transcriptional activation but not proliferation in midbrain astrocytes. (**A** and **B**) IHC analysis of GFAP staining in the SNpc in indicated genotypes 3 days after MPTP treatment (scale bar = 200 μm). (**C** and **D**) Flow cytometric analysis of GLAST^+^ astrocytes in midbrain homogenates derived from indicated genotypes 3 days after MPTP treatment. (**E** and **F**) qRT-PCR analysis of indicated genes in midbrain homogenates derived from astrocyte-specific *Ripk3* knockouts (**E**) or astrocyte-specific *Ripk3*-overexpressing (**F**) mice 3 days after MPTP treatment. (**G** and **H**) Schematic of inducible RIPK3 activation system (**G**) and stereotactic delivery of dimerization drug into the ventral midbrain (**H**). (**I**) qRT-PCR analysis of indicated genes in midbrain homogenates derived from *Ripk3*-2xFV^fl/fl^ *Aldh1l1*-Cre^+^ mice 24 hours following administration of B/B homodimerizer or vehicle control. *n* = 3–8 mice/group (**A** and **B**), 5 mice/group (**C** and **D**), 6–9 mice/group (**E**), 7–8 mice/group (**F**), 5 mice/group (**I**). Data are represented as mean values with scatterplots depicting individual biological replicate values. Comparisons via 2-tailed Student’s *t* test (**D**) or 2-way ANOVA with Holm-Šídák multiple-comparison test (**B**, **E**, **F**, and **I**). *P < 0.05, **P < 0.01, ***P < 0.001. **G** and **H** were created with Biorender.com.

Given these findings, we next questioned how RIPK3 signaling influences the phenotype of astrocytes in the setting of MPTP administration. Immunohistochemical (IHC) staining of SNpc sections revealed increased glial fibrillary acidic protein (GFAP) staining in MPTP-treated control animals, consistent with astrocyte activation, and this effect was blocked in *Ripk3*^fl/fl^ *Aldh1l1*^Cre+^ mice (Figure 2, A and B). To test whether enhanced GFAP staining indicated proliferative astrogliosis, we performed flow cytometric analysis of astrocytes in the midbrain of MPTP-treated animals, which revealed no differences in glutamate aspartate transporter–positive (GLAST^+^) astrocytes between genotypes (Figure 2, C and D). These data suggested that enhanced GFAP staining was not due to increased numbers of astrocytes following MPTP administration, but rather a change in the astrocyte activation status. To test this idea, we performed quantitative reverse-transcription PCR (qRT-PCR) analysis of a panel of transcripts that we and others have shown to be associated with neurotoxic astrocyte activation in models of Parkinson’s disease (14, 31, 32). We observed upregulation of 10 out of 14 transcripts in our analysis panel in midbrain homogenates derived from MPTP-treated littermate controls, while this activation signature was essentially abolished in *Ripk3*^fl/fl^ *Aldh1l1*^Cre+^ mice (Figure 2E). In contrast, MPTP-treated *Mlkl*^–/–^ mice showed equivalent levels of inflammatory transcript expression in the midbrain (Supplemental Figure 2A). We further verified a lack of MLKL phosphorylation in midbrain homogenates of MPTP-treated mice using ELISA, suggesting that MLKL is not activated in this region in the subacute MPTP model (Supplemental Figure 2B). These data suggest that astrocytic RIPK3 signaling promotes an inflammatory transcriptional state in the midbrain following MPTP treatment, independently of MLKL and necroptosis.

We next more carefully assessed this idea by using a mouse line expressing RIPK3 fused to 2 FKBP^F36V^ domains that facilitate enforced oligomerization following treatment with a dimerization drug. This protein is expressed in a cell type–specific manner under the control of a lox-STOP-lox element in the *Rosa26* locus, while the endogenous *Ripk3* locus is left intact. Thus, this mouse line can be used as a cell type–specific overexpression system while also facilitating forced chemogenetic activation of RIPK3 in cell types of interest in vivo (12, 13, 33). We first questioned whether simple overexpression of RIPK3 in astrocytes would enhance the inflammatory transcriptional signature that occurs following MPTP administration. We observed that 4 neurotoxic astrocyte-associated transcripts exhibited augmented upregulation following MPTP administration in *Ripk3*-2xFV^fl/fl^ *Aldh1l1*^Cre+^ mice, including *Ccl5*, *Cd14*, *Cxcl10*, and *Psmb8*, while 2 others exhibited trends toward increased expression that did not reach statistical significance (*Cd109*, *H2-D1*) (Figure 2F). To assess whether activation of astrocytic RIPK3 was sufficient to induce an inflammatory gene signature, we enforced RIPK3 activation in astrocytes via stereotactic delivery of B/B homodimerizer to the ventral midbrain of *Ripk3*-2xFV^fl/fl^ *Aldh1l1*^Cre+^ mice. B/B homodimerizer binds in a multivalent fashion to the FKBP^F36V^ domains of RIPK3-2xFV proteins, driving their oligomerization, which is sufficient to induce RIPK3 kinase activity in the absence of any other stimulus (34, 35) (Figure 2, G and H). Enforced activation of RIPK3 in midbrain astrocytes in vivo resulted in induced expression of several neurotoxic astrocyte-associated transcripts, including *Cd14*, *Emp1*, *Gbp2*, *Lcn2*, *S100a10*, and *Srgn* (Figure 2I). Together, these data show that activation of RIPK3 in midbrain astrocytes drives their activation and the establishment of an inflammatory transcriptional signature.

### Astrocytic RIPK3 signaling has minimal impact on microglial activation in the MPTP model.

**Figure 3:**
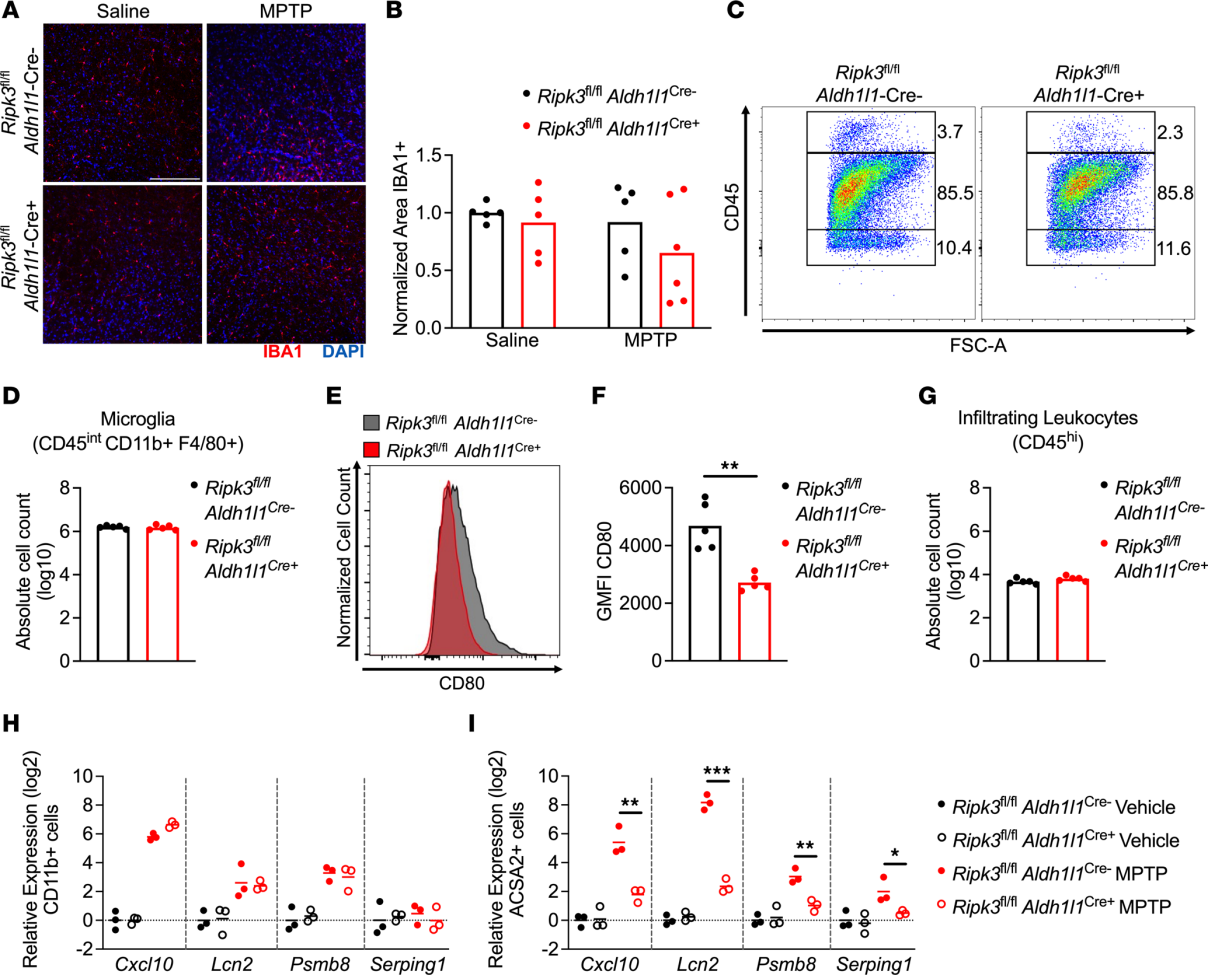
Astrocytic RIPK3 signaling has minimal impact on microglial activation in the MPTP model. (**A** and **B**) IHC analysis of IBA1 staining in the SNpc in indicated genotypes 3 days post-MPTP treatment (scale bar = 200 μm). (**C**) Representative flow cytometric plot depicting leukocyte populations in midbrain homogenates derived from indicated genotypes 3 days post-MPTP treatment. (**D**) Quantification of absolute numbers of microglia derived from flow cytometric analysis. (**E** and **F**) Representative histogram (**E**) and quantification of geometric mean fluorescence intensity (GMFI) (**F**) derived from analysis of CD80 expression on microglial populations in **D**. (**G**) Quantification of absolute numbers of CD45^hi^ leukocytes derived from flow cytometric analysis. (**H** and **I**) qRT-PCR analysis of indicated genes in sorted microglia (**H**) or astrocytes (**I**) derived from astrocyte-specific *Ripk3*-knockout mice 3 days post-MPTP treatment. *n* = 5–6 mice/group (**A** and **B**), 5 mice/group (**C**–**G**), 3 mice/group (**H** and **I**). Data are represented as mean values with scatterplots depicting individual biological replicate values. Comparisons via 2-tailed *t* test (**D**, **F**, and **G**) or 2-way ANOVA with Holm-Šídák multiple-comparison test (**B**, **H**, and **I**). *P < 0.05, **P < 0.01, ***P < 0.001.

We next questioned whether the reduced expression of inflammatory genes observed in mice lacking astrocytic RIPK3 was associated with cell-nonautonomous effects on other cell types in the setting of MPTP treatment. We thus performed IHC staining for ionized calcium-binding adapter molecule 1 (IBA1), a marker of myeloid cells that largely labels microglia in the setting of sterile neurodegeneration (36, 37). This analysis revealed no differences in the overall coverage of IBA1 staining in the midbrain in *Ripk3*^fl/fl^ *Aldh1l1*^Cre+^ mice compared with littermate controls (Figure 3, A and B). To assess changes to immune cells more carefully, we next performed flow cytometric analysis of leukocytes derived from the midbrain of MPTP-treated mice. This revealed essentially identical frequencies of CD45^int^CD11b^+^F4/80^+^ microglia between genotypes (Figure 3, C and D), suggesting a lack of difference in microglial proliferation. Despite this, microglia derived from MPTP-treated *Ripk3*^fl/fl^ *Aldh1l1*^Cre+^ mice exhibited diminished expression of the costimulatory molecule CD80 compared with controls (Figure 3, E and F), consistent with a less inflammatory phenotype. We also observed very low frequencies of CD45^hi^ infiltrating peripheral immune cells in the MPTP model (Figure 3C), the overall numbers of which did not differ by genotype (Figure 3G). To more explicitly test which cell types were driving differences in the midbrain transcriptional response in *Ripk3*^fl/fl^ *Aldh1l1*^Cre+^ animals, we sorted CD11b^+^ myeloid cells (primarily microglia, given very low levels of infiltrating leukocytes) and ACSA2^+^ astrocytes and assessed transcript levels of a subset of highly differentially expressed inflammatory genes identified in our studies using midbrain homogenates. We observed significantly diminished expression of *Cxcl10*, *Lcn2*, *Psmb8*, and *Serping1* in sorted astrocytes but not in sorted microglia derived from MPTP-treated *Ripk3*^fl/fl^ *Aldh1l1*^Cre+^ mice compared with controls (Figure 3, H and I). These data suggest that astrocytic RIPK3 signaling following MPTP administration likely induces neuroinflammation primarily through cell-intrinsic mechanisms, with only modest cell-nonautonomous effects on microglia.

### Astrocytic RIPK3 activation drives a transcriptomic state associated with inflammation and neurodegeneration in the midbrain.

**Figure 4:**
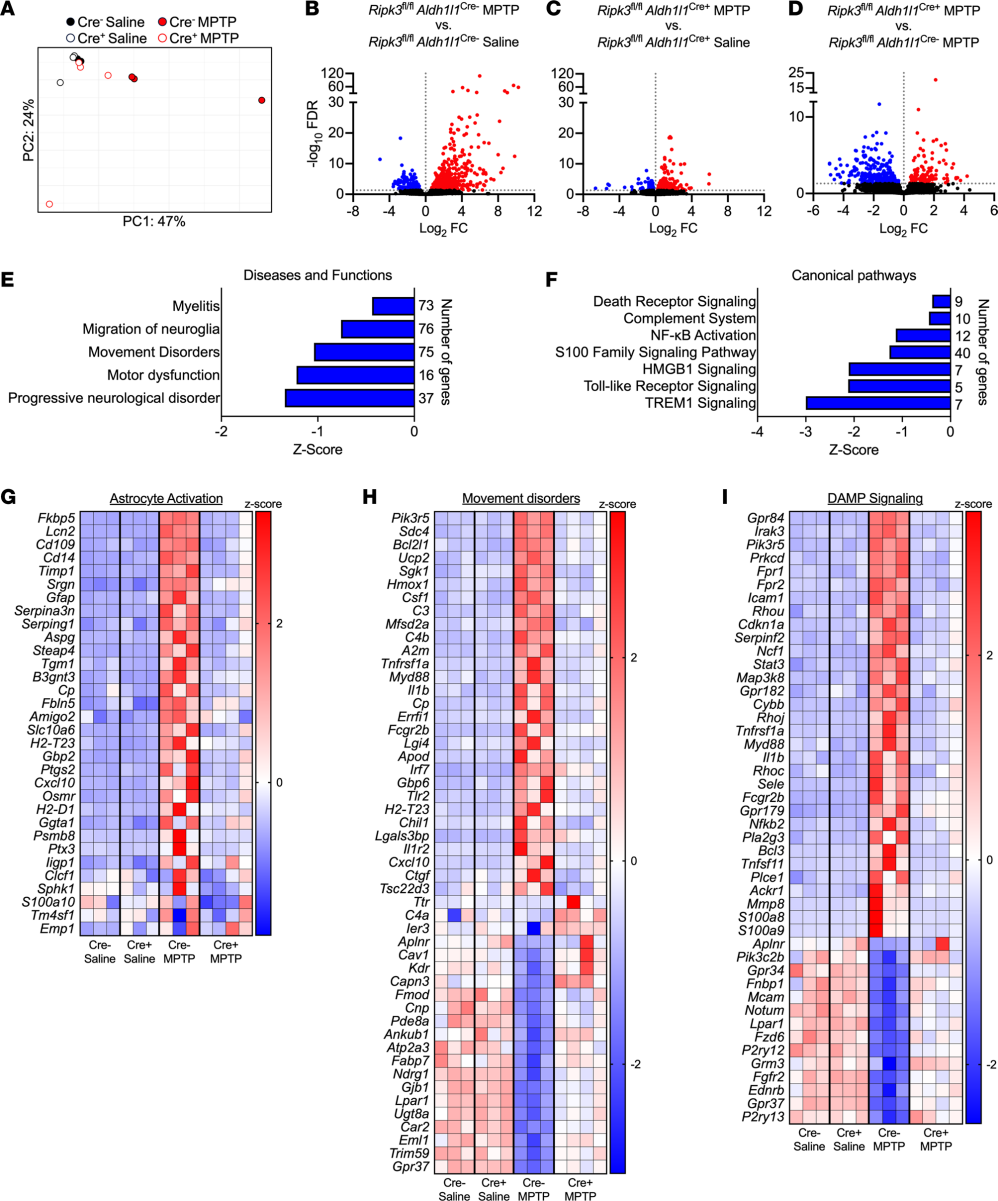
Astrocytic RIPK3 activation drives a transcriptomic state associated with inflammation and neurodegeneration in the midbrain. (**A**–**I**) Midbrains were harvested from mice of indicated genotypes 3 days posttreatment with MPTP or saline and subjected to bulk RNA-Seq. (**A**) Principal component analysis demonstrating separation of treatment groups and genotypes in the RNA-Seq data set. (**B**–**D**) Volcano plots showing differentially expressed genes derived from indicated comparisons. Data points in red are genes exhibiting upregulated expression, while those in blue exhibit downregulated expression. Genes with an FDR < 0.05 were considered significant. (**E** and **F**) Selected significantly enriched disease and function terms (**E**) or canonical pathways (**F**) derived from Ingenuity Pathway Analysis comparing Cre^–^ versus Cre^+^ MPTP-treated groups. (**G**–**I**) Heatmaps showing significantly differentially expressed genes for selected pathways. *n* = 3–4 mice/group in all panels.

To characterize how astrocytic RIPK3 shapes the neuroinflammatory state of the brain more thoroughly in the MPTP model, we also performed bulk RNA sequencing (RNA-Seq) of isolated midbrain tissues derived from *Ripk3*^fl/fl^ *Aldh1l1*^Cre+^ mice and littermate controls. Principal component analysis revealed distinct separation of MPTP-treated control animals along PC1, while MPTP-treated conditional knockouts largely clustered with vehicle-treated animals of both genotypes (Figure 4A). Further analysis revealed a robust transcriptional response to MPTP in midbrain tissues of littermate control animals, including 452 significantly upregulated genes and 145 significantly downregulated genes (Figure 4B) compared with vehicle-treated controls. This transcriptional response was blunted in *Ripk3*^fl/fl^ *Aldh1l1*^Cre+^ mice, which exhibited only 195 significantly upregulated genes and 120 significantly downregulated genes compared with genotype-matched vehicle-treated animals (Figure 4C), suggesting that astrocytic RIPK3 signaling drives a major portion of the tissue-wide transcriptional response to MPTP-induced neuronal cell death. In support of this idea, comparison of differentially expressed genes (DEGs) within MPTP-treated groups revealed 120 genes with significantly higher expression and 252 genes with significantly lower expression in conditional knockouts compared with littermate controls (Figure 4D).

To better understand the functional relevance of these transcriptomic profiles, we performed Ingenuity Pathway Analysis (IPA; QIAGEN) of genes differentially expressed between genotypes in MPTP-treated animals. This revealed significant enrichment of several disease and function terms with relevance to our study, including Progressive Neurological Disorder, Movement Disorders, and others (Figure 4E). Comparisons of differentially regulated canonical pathways showed significant enrichment of pathways relating to programmed cell death and inflammation, as expected (Figure 4F). Notably, terms related to DAMP signaling were also highly enriched, including signaling by HMGB1 and S100 family proteins, both of which are factors released by dying and damaged cells that induce inflammation. Further analysis revealed significant upregulation of genes associated with astrocyte activation (Figure 4G), consistent with our previous qRT-PCR analysis. Comparisons of individual gene expression profiles for 2 selected IPA terms (Movement Disorders and DAMP signaling) (Figure 4, H and I) revealed dozens of significant DEGs for both terms, characterized by a mix of both up- and downregulated gene expression. Together, our RNA-Seq analysis reveals a central role for astrocytic RIPK3 in promoting gene expression associated with neurodegeneration and neuroinflammation in the midbrain. Our findings also suggest a strong link between DAMP signaling and RIPK3-dependent neuroinflammation.

### Secreted factors from dying neurons drive RIPK3-dependent astrocyte activation.

**Figure 5:**
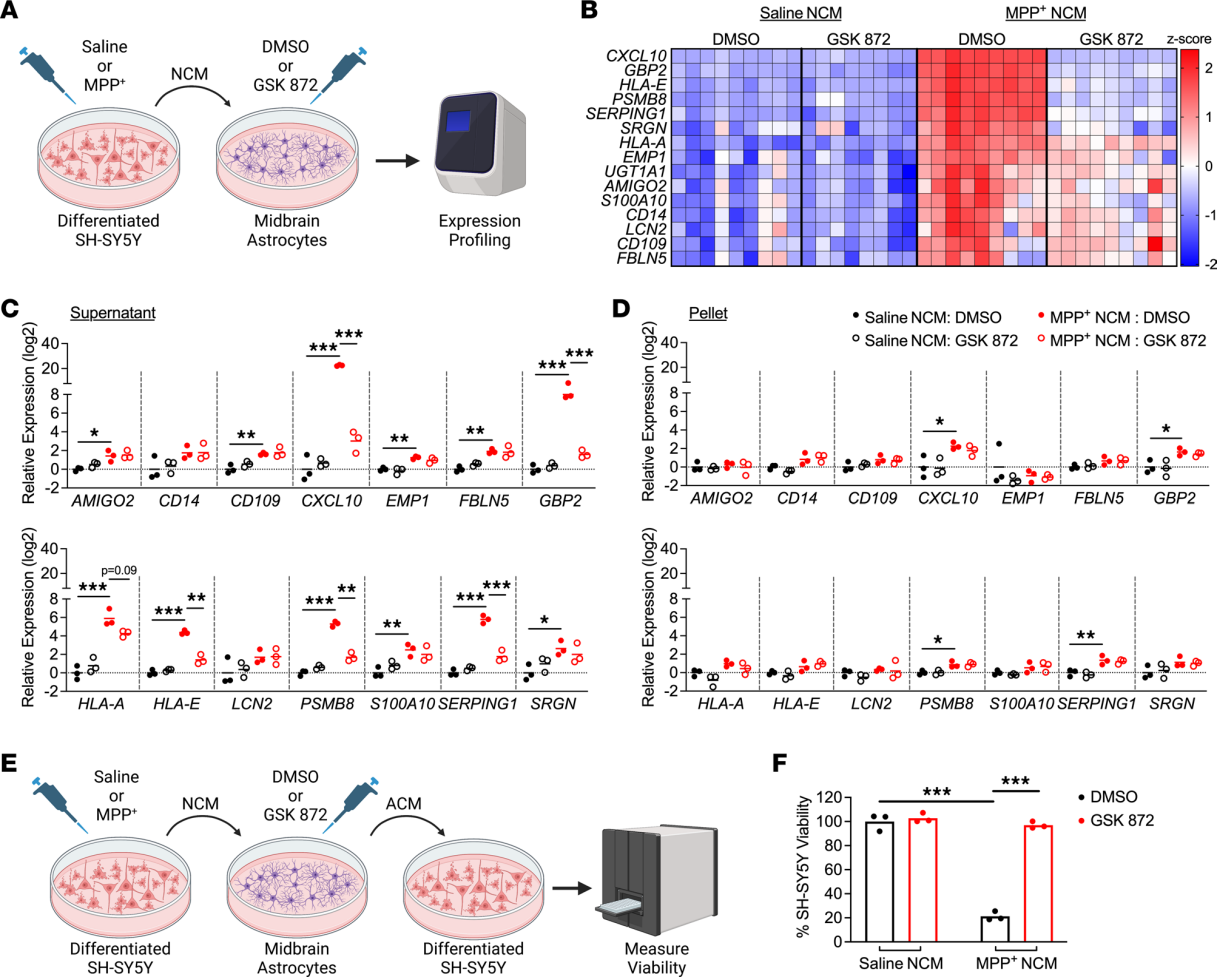
Secreted factors from dying neurons drive RIPK3-dependent astrocyte activation. (**A**) Schematic of experimental design for DAMP transfer experiments. Differentiated SH-SY5Y cells were treated with MPP^+^ or saline for 24 hours, and medium (NCM) was then transferred to cultures of primary human midbrain astrocytes. Astrocytes were treated with NCM in the presence of GSK872 or control for 24 hours prior to qRT-PCR profiling. (**B**) Heatmap showing expression of astrocyte activation–associated genes in astrocyte cultures treated as in **A**. (**C** and **D**) qRT-PCR profiling of indicated genes in astrocytes treated for 24 hours with clarified NCM supernatants (**C**) or pelleted SH-SY5Y debris (**D**). (**E**) Schematic of experimental design for neurotoxicity assay. Astrocytes were treated with NCM as in **A** for 24 hours. Astrocytes were then washed and media replaced for another 24 hours. This new astrocyte-conditioned medium (ACM) was then transferred to fresh SH-SY5Y cells for cell viability measurement. (**F**) CellTiter-Glo analysis of SH-SY5Y viability 24 hours following treatment with ACM derived from indicated conditions. *n* = 9 cultures/group (**A**), 3 cultures/group (**C**, **D**, and **F**). Data are represented as mean values with scatterplots depicting individual biological replicate values. All comparisons via 2-way ANOVA with Holm-Šídák multiple-comparison test. *P < 0.05, **P < 0.01, ***P < 0.001. **A** and **E** were created with Biorender.com.

Given the strong representation of DAMP signaling in our transcriptomic analysis, we questioned whether factors released from dying neurons were important for driving RIPK3-mediated astrocyte activation. To test this, we treated differentiated SH-SY5Y neuroblastoma cells, a commonly used model of catecholaminergic neurons (38), with the toxic MPTP metabolite MPP^+^ (5 mM) for 24 hours, which resulted in around 50% cell death (Supplemental Figure 3A). We harvested the neuronal conditioned media (NCM) from these cells, which contained DAMPs and other factors released from dying SH-SY5Y cells, and added it to primary human midbrain astrocyte cultures at a ratio of 1:1 with normal astrocyte culture media (Figure 5A). NCM-treated astrocytes were also treated with the RIPK3 inhibitor GSK872 or DMSO vehicle. qRT-PCR analysis of a panel of top DEGs associated with astrocyte activation identified in our in vivo transcriptomic profiling revealed robust induction of inflammatory gene expression in midbrain astrocyte cultures treated with NCM derived from MPP^+^-treated SH-SY5Y cultures, hereafter referred to as MPP^+^ NCM (Figure 5B), following 24 hours of stimulation. However, pharmacologic inhibition of RIPK3 signaling in astrocytes largely prevented this effect.

After these observations, we recognized that our NCM preparations may have contained debris and floating “corpses” from dead SH-SY5Y cells. To assess whether soluble factors or dead cell–associated material was the primary driver of RIPK3-dependent astrocyte activation in our experiments, we carefully fractionated NCM samples to pellet out cellular material from soluble factors in the media. Application of either clarified supernatant (Figure 5C) or resuspended pellet material (Figure 5D) from MPP^+^-treated SH-SY5Y cells to midbrain astrocyte cultures revealed that clarified supernatants stimulated expression of many inflammatory genes in astrocytes in a largely RIPK3-dependent manner. In contrast, pellet-derived material was only minimally stimulatory, and this stimulation was RIPK3 independent. We also verified that exposure to residual MPP^+^ in NCM was not the primary driver of astrocyte activation, as direct application of MPP^+^ to midbrain astrocyte cultures did not result in either cell death or upregulation of inflammatory gene expression (Supplemental Figure 3, B and C). As we and others have shown that RIPK3 promotes inflammatory gene expression largely through NF-κB activation (14, 33, 39), we also verified that clarified MPP^+^ NCM supernatants induced NF-κB activation in astrocytes in a RIPK3-dependent manner (Supplemental Figure 4A) and that blockade of NF-κB signaling with the pharmacologic agent JSH-23 greatly suppressed the stimulatory effect of MPP^+^ NCM (Supplemental Figure 4B).

We next wanted to verify that inflammatory gene expression in our system corresponded to a functional readout of astrocyte activation. We thus assessed whether exposure to dying neuron–derived factors would confer neurotoxic activity to astrocytes. We first treated human midbrain astrocytes for 24 hours with MPP^+^ NCM with or without RIPK3 inhibitor (and respective controls), then washed the cells and replaced the astrocyte medium to remove residual MPP^+^. We then cultured astrocytes for an additional 24 hours and collected their conditioned media (ACM), which was then added to fresh cultures of SH-SY5Y cells at a 1:1 ratio with normal SH-SY5Y media (Figure 5E). We observed that astrocytes maintained transcriptional activation for at least 24 hours following this wash step, verifying that astrocytes remain activated after removal of MPP^+^ NCM in this paradigm (Supplemental Figure 5). ACM derived from MPP^+^ NCM–treated astrocytes induced around 80% cell death in fresh SH-SY5Y cultures after 24 hours, whereas this neurotoxic activity was completely abrogated when astrocytic RIPK3 signaling was inhibited (Figure 5F). Together, these data show that soluble factors released from dying neuron-like cells are sufficient to induce inflammatory transcription and neurotoxic activity in midbrain astrocytes and that this process requires, to a large degree, cell-intrinsic RIPK3 activity within astrocytes.

### RIPK3 activation is sufficient to induce astrocyte-mediated killing of primary neurons.

**Figure 6:**
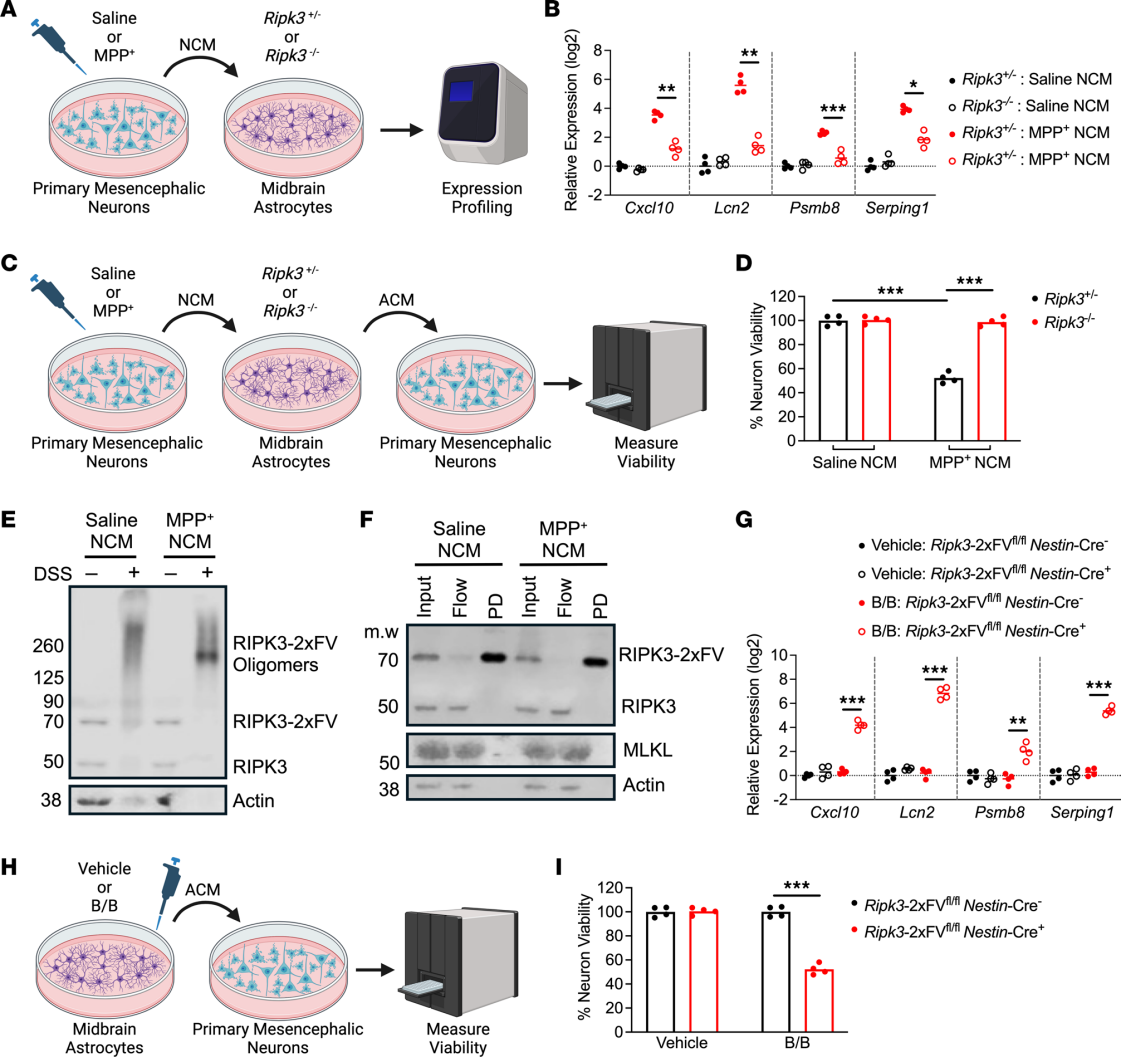
RIPK3 activation is sufficient to induce astrocyte-mediated killing of primary neurons. (**A**) Schematic of experimental design for DAMP transfer experiments. (**B**) qRT-PCR profiling of indicated genes in astrocytes treated for 24 hours with clarified NCM supernatants. (**C**) Schematic of experimental design for neurotoxicity assay. (**D**) CellTiter-Glo analysis of neuron viability 24 hours following treatment with ACM derived from indicated conditions. (**E** and **F**) Western blot analysis of indicated proteins in astrocytes expressing FLAG-tagged RIPK3 following 24 hours of treatment with NCM and DSS cross-linking (**E**) or bead-mediated FLAG pulldown (**F**). (**G**) qRT-PCR profiling of indicated genes in astrocytes of indicated genotypes treated for 24 hours with B/B homodimerizer. (**H**) Schematic of experimental design for neurotoxicity assay in which astrocytes expressing (or not) RIPK3-2xFV were treated with B/B homodimerizer or vehicle solution for 24 hours. Astrocytes were then washed and media replaced for another 24 hours. ACM was then transferred to WT primary neurons for cell viability measurement. (**I**) CellTiter-Glo analysis of viability in WT neurons 24 hours following treatment with ACM derived from indicated conditions. *n* = 4 cultures/per group in all panels. Data are represented as mean values with scatterplots depicting individual biological replicate values. All comparisons via 2-way ANOVA with Holm-Šídák multiple-comparison test. *P < 0.05, **P < 0.01, ***P < 0.001. **A**, **C**, and **H** were created with Biorender.com.

Although our results using the SH-SY5Y cell line were promising, we next sought to recapitulate these findings with bona fide primary neuron cultures. We thus treated primary murine mesencephalic neuron cultures with MPP^+^ or saline to generate NCM, similar to our previous experiments with SH-SY5Y cells. NCM was applied to primary murine midbrain astrocytes derived from *Ripk3*^–/–^ mice or their *Ripk3*^+/–^ littermates (Figure 6A). Expression profiling revealed greatly enhanced expression of inflammatory genes in MPP^+^ NCM–treated control astrocytes, while this effect was significantly blunted in astrocytes lacking *Ripk3* expression (Figure 6B). To test whether this RIPK3-dependent gene expression was associated with neurotoxic activity, we generated ACM samples from this paradigm and applied them to fresh cultures of primary mesencephalic neurons (Figure 6C). Primary neurons exposed to the conditioned medium of MPP^+^ NCM–treated *Ripk3*^+/–^ astrocytes exhibited significantly diminished viability, while this effect was lost when astrocytes lacked *Ripk3* expression (Figure 6D). To verify that treatment with MPP^+^ NCM was sufficient to drive RIPK3 activation, we used primary midbrain astrocyte cultures expressing the chimeric RIPK3-2xFV protein, which contains a FLAG-tag (12, 13), under the *Nestin* promoter (which drives expression in astrocyte cultures derived from neonates) in order to facilitate molecular biological analysis. Treatment of RIPK3-2xFV–expressing midbrain astrocytes with MPP^+^ NCM resulted in robust RIPK3 activation, as evidenced by the abundance of high–molecular weight RIPK3 oligomers in samples subjected to DSS cross-linking (Figure 6E). To assess whether these complexes interacted with MLKL, we pulled down RIPK3 following exposure to NCM using beads coated with anti-FLAG antibodies. While we observed highly efficient pulldown of RIPK3, we saw no evidence of interaction with MLKL in pulldown samples (Figure 6F), consistent with the idea that changes to astrocyte activation in our model are not due to MLKL activation and necroptosis. We separately verified that MPP^+^ NCM did not induce cell death in primary midbrain astrocytes, nor did it induce MLKL phosphorylation (Supplemental Figure 6, A and B). We also tested whether direct chemogenetic activation of RIPK3 was sufficient to reproduce our phenotype by treating RIPK3-2xFV–expressing astrocytes with B/B homodimerizer. This treatment resulted in robust induction of inflammatory gene expression in *Nestin*-Cre^+^ cultures but not in cultures lacking transgene expression (*Nestin*-Cre^–^) (Figure 6G). Finally, we also generated ACM from astrocytes treated in this paradigm (Figure 6H) and tested for neurotoxic activity on primary mesencephalic neurons, which revealed that chemogenetic activation of astrocytic RIPK3 was also sufficient to induce neurotoxicity (Figure 6I). Together, these data support our findings that necroptosis-independent RIPK3 activation is sufficient to drive inflammatory and neurotoxic activity in midbrain astrocytes.

### DAMP signaling via RAGE drives inflammatory activation in midbrain astrocytes.

**Figure 7:**
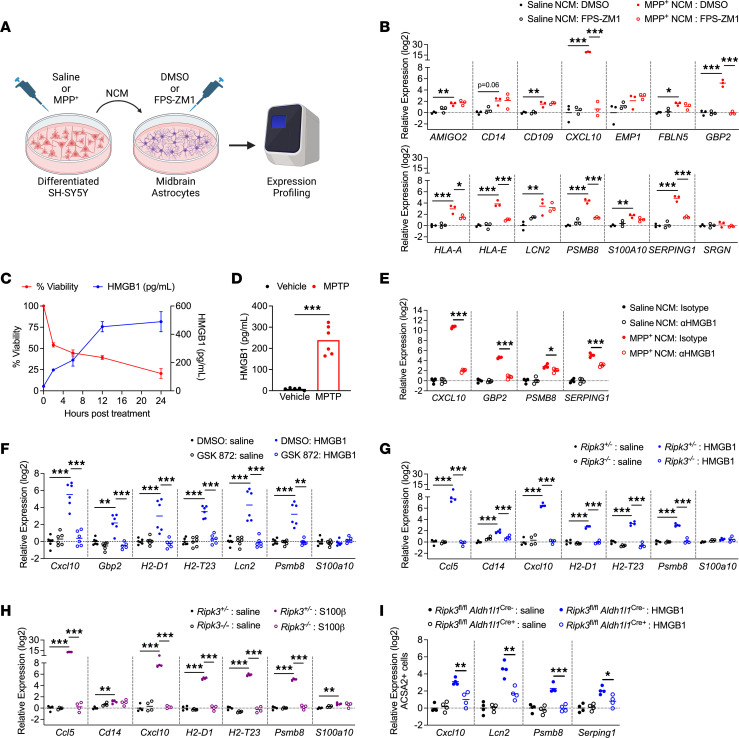
DAMP signaling via RAGE drives inflammatory activation in midbrain astrocytes. (**A**) Schematic of experimental design for DAMP transfer experiments. Astrocytes were treated with NCM in the presence of FPS-ZM1 or control for 24 hours prior to qRT-PCR profiling. (**B**) qRT-PCR profiling of indicated genes in astrocytes treated for 24 hours with NCM derived from indicated conditions. (**C** and **D**) ELISA analysis of HMGB1 protein levels in supernatants of SH-SY5Y cells treated with MPP^+^ (**C**) or midbrain homogenates from WT mice 3 days post-MPTP treatment (**D**). *n* = 4–8 replicates per time point in **C**. (**E**) qRT-PCR profiling of indicated genes in human midbrain astrocytes treated for 24 hours with NCM derived from indicated conditions in the presence of neutralizing antibodies against HMGB1 (1 μg/mL) or an isotype control antibody. (**F**–**H**) qRT-PCR analysis of indicated genes in WT murine midbrain astrocytes (**F**) or midbrain astrocytes derived from indicated genotypes (**G** and **H**) 24 hours following treatment with recombinant HMGB1 (**F** and **G**) or S100β (**H**). (**I**) qRT-PCR analysis of indicated genes in ACSA2^+^ astrocytes sorted via MACS from brains of mice 24 hours following ICV administration of HMGB1 (200 ng). *n* = 3 cultures/group (**B**), 8 cultures/group for viability data and 2–4 cultures per group for HMGB1 expression (**C**), 5–6 mice/group (**D**), 6 cultures/group (**E**), 4 cultures/group (**F** and **G**), and 4 mice/group (**H**). Data are represented as mean values with scatterplots depicting individual biological replicate values, except in **C**, where data are represented as mean values ± SEM. Comparisons via 2-tailed *t* test (**D**) or 2-way ANOVA with Holm-Šídák multiple-comparison test (**B** and **E**–**I**). *P < 0.05, **P < 0.01, ***P < 0.001. **A** was created with Biorender.com.

We next sought to more precisely identify specific DAMP signals that stimulate midbrain astrocyte activation. Our transcriptomic analysis revealed that both HMGB1 and S100 family signaling were highly enriched in an astrocytic RIPK3-dependent manner in the midbrain following MPTP treatment. As both of these DAMPs stimulate a common receptor, RAGE, we assessed whether RAGE was required for astrocyte activation following exposure to MPP^+^ NCM. We thus treated human midbrain astrocyte cultures with MPP^+^ or control NCM, along with the RAGE inhibitor FPS-ZM1, for 24 hours and performed qRT-PCR profiling (Figure 7A). Blockade of RAGE in astrocytes substantially reduced MPP^+^ NCM–induced transcriptional activation, effectively preventing upregulation of 6 out of 11 astrocyte activation–associated transcripts (Figure 7B). Based on these findings, we verified that the RAGE ligand HMGB1 was, in fact, released by SH-SY5Y cells following induction of cell death by MPP^+^ (Figure 7C). We also observed significant accumulation of HMGB1 protein in midbrain homogenates of mice treated with MPTP (Figure 7D), verifying that induction of dopaminergic cell death results in the release of RAGE ligands in vivo. We further verified that RAGE ligands drive astrocyte activation in our model by treating midbrain astrocytes with NCM in the presence of HMGB1 neutralizing antibodies, which significantly blunted the transcriptional activation induced by MPP^+^ NCM (Figure 7E).

To assess whether RAGE ligands induced astrocyte activation in a RIPK3-dependent manner, we next treated primary midbrain astrocytes with recombinant DAMPs and profiled gene expression. Strikingly, we observed that stimulation of murine midbrain astrocytes with HMGB1 induced robust transcriptional activation that was blocked in the presence of GSK872 (Figure 7F). As a complementary approach, we also generated midbrain astrocyte cultures from *Ripk3^–/–^* mice (and heterozygous littermate controls) and stimulated with RAGE ligands. Treatment with either HMGB1 (Figure 7G) or S100β (Figure 7H) induced inflammatory transcript expression in control but not *Ripk3*^–/–^ cultures. To verify that HMGB1 could drive RIPK3-dependent astrocyte activation in vivo, we performed intracerebroventricular (ICV) administration of recombinant HMGB1 in *Ripk3*^fl/fl^ *Aldh1l1*^Cre+^ mice and littermate controls. We then sorted ACSA2^+^ astrocytes via MACS 24 hours following HMGB1 treatment. While ICV delivery of HMGB1 robustly induced transcriptional activation in control astrocytes, this effect was significantly blunted in astrocytes lacking *Ripk3* expression (Figure 7I). Together, these data support a model in which dying neurons release DAMPs that induce inflammatory astrocyte activation through activation of astrocytic RAGE, which in turn drives transcriptional activation via RIPK3 signaling.

### Activation of RIPK3 by DAMP signaling drives pathogenic functional changes in midbrain astrocytes.

**Figure 8:**
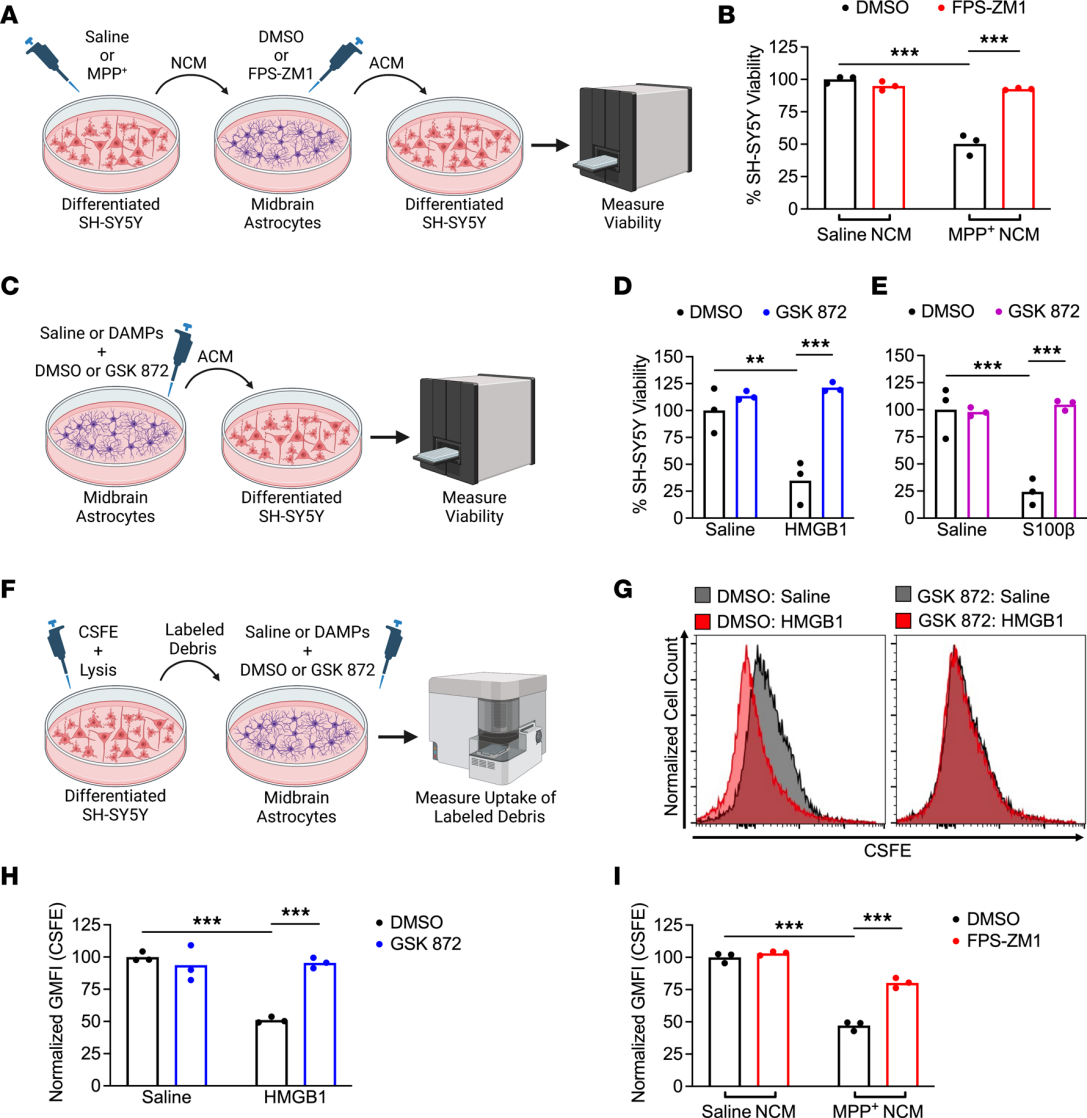
Activation of RIPK3 by DAMP signaling drives pathogenic functional changes in midbrain astrocytes. (**A**) Schematic of experimental design for neurotoxicity experiments. Astrocytes were treated with NCM in the presence of FPS-ZM1 or control for 24 hours. ACM was then transferred to fresh SH-SY5Y cells for cell viability measurement. (**B**) CellTiter-Glo analysis of SH-SY5Y viability 24 hours following treatment with ACM derived from indicated conditions. (**C**) Schematic showing treatment of primary human midbrain astrocytes with recombinant DAMPs for 24 hours prior to transfer of ACM to SH-SY5Y cultures. (**D** and **E**) CellTiter-Glo analysis of SH-SY5Y viability 24 hours following treatment with ACM derived from indicated conditions. (**F**) Schematic showing generation and transfer of CSFE-labeled neuronal debris to midbrain astrocytes treated with recombinant DAMPs with or without GSK872. Astrocytes were cultured in the presence of labeled debris for 24 hours. (**G** and **H**) Representative histograms (**G**) and quantification of GMFI (**H**) of CSFE signal in astrocytes treated as in **F**. (**I**) GMFI of CSFE internalization in astrocytes treated as in **F** but with NCM rather than recombinant DAMPs and FPS-ZM1 rather than GSK872. *n* = 3 cultures/group in all panels. Data are represented as mean values with scatterplots depicting individual biological replicate values. All comparisons via 2-way ANOVA with Holm-Šídák multiple-comparison test. **P < 0.01, ***P < 0.001. **A**, **C**, and **F** were created with Biorender.com.

To verify that the transcriptional effects of DAMP signaling affected astrocyte function, we collected ACM from astrocytes treated for 24 hours with MPP^+^ NCM with or without RAGE inhibitor (and respective controls) and applied the ACM to fresh cultures of SH-SY5Y cells (Figure 8A). ACM derived from MPP^+^ NCM-treated astrocytes robustly induced cell death in fresh SH-SY5Y cultures, while this neurotoxic activity was completely abrogated when astrocytic RAGE signaling was inhibited (Figure 8B). We also observed conferral of neurotoxic activity following direct stimulation of astrocytes with recombinant DAMPs (Figure 8C), including HMGB1 (Figure 8D) and S100β (Figure 8E). However, this neurotoxic activity was also abrogated when RIPK3 signaling was blocked, further supporting a role for a RAGE/RIPK3 axis in promoting neurotoxic astrocyte activation. This neurotoxic activity was not due to residual recombinant DAMPs in ACM, as direct application of either DAMP ligand to SH-SY5Y cells did not result in cell death (Supplemental Figure 7). As previous work has shown that neurotoxic astrocytes downregulated key homeostatic functions such as phagocytosis (14, 31), we also exposed midbrain astrocyte cultures to labeled debris generated from SH-SY5Y cells and measured phagocytic uptake of debris via flow cytometry (Figure 8F). Direct stimulation of astrocytes with HMGB1 resulted in a significant reduction in uptake of CSFE-labeled debris, while this suppression of phagocytic function was blocked in the presence of a RIPK3 inhibitor (Figure 8, G and H). We also observed that MPP^+^ NCM similarly reduced astrocytic phagocytosis in a RIPK3-dependent fashion (Figure 8I). These data further support the notion that DAMPs emanating from dying neurons alter astrocytic function via activation of RIPK3 signaling.

## Discussion

Our study defines a potentially previously unknown role for neuronal DAMPs in promoting neurotoxic astrocyte activation. This effect was mediated by RIPK3-mediated transcriptional activation, an effect that occurred independently of the necroptotic executioner protein MLKL. Mechanistically, we found that astrocytic RAGE signaling was required for astrocyte activation downstream of DAMP exposure, and this RAGE/RIPK3 signaling axis promoted inflammatory transcription and neurotoxic functional activity. Intriguingly, these results suggest that neuronal death, itself, potentiates a feed-forward process of astrocyte activation and further neuronal cell death. These findings highlight an important mechanism of neuron-glia crosstalk in the pathogenesis of neurodegeneration.

DAMPs have previously been implicated as drivers of inflammation in a broad variety of disorders, including neurodegeneration, ischemic stroke, autoimmunity, cardiovascular disease, and others (40–46). RAGE ligands, in particular, have been associated with neurodegenerative disease and have been the target of preclinical therapeutic development. For example, S100β levels in serum and cerebrospinal fluid has been shown to correlate with disease severity in Parkinson’s disease (22, 47). Mice deficient in S100β are also resistant to MPTP-driven neurodegeneration (22), consistent with a role for this molecule in perpetuating neuronal cell death. Similarly, antibody-mediated neutralization of HMGB1 has been shown to attenuate glial cell activation and prevent neuron loss in models of both Alzheimer’s disease and Parkinson’s disease (21, 48). Despite these findings, other groups have also described neuroprotective functions for RAGE ligands (49), including stimulation of neurotrophic growth factor expression in amyotrophic lateral sclerosis (50), suppression of amyloidosis (51), and direct antiapoptotic effects in neurons (52, 53). These complex effects appear to be highly context dependent, differing by cell type, disease state, and even DAMP concentration (52, 54, 55). Our data support a pathogenic role for RAGE signaling in the promotion of neurotoxic astrocyte activation.

Astrocytes express RAGE and other DAMP sensors, although cell type–specific functions for DAMP signaling in astrocytes have not been thoroughly studied (56). Existing studies suggest that astrocytic RAGE signaling is pathogenic, on balance (57–59). In Huntington’s disease, RAGE-positive astrocytes have been shown to have high levels of nuclear NF-κB (58), consistent with a role for this pathway in promoting inflammatory astrocyte activation. Diminished levels of HMGB1 following berberine treatment also correlated with diminished astrocyte activation in a model of sepsis (60). Astrocytes are also major sources of RAGE ligands, particularly S100β, and much work to date has focused on autocrine RAGE signaling in astrocytes as a result (61–63). We took advantage of the MPTP model, which induces death selectively in neurons but not astrocytes (64), as well as serial culture systems to more directly assess the impact of paracrine RAGE signaling on astrocyte activation and function. Our study suggests that DAMPs released from dying neurons potently induce inflammatory astrocyte activation via RAGE, driving neurotoxic activation and perpetuating further neuronal cell death. These findings identify RAGE as a promising target for modulating astrocytic responses to neuronal cell death during neurodegenerative disease.

RIPK3 signaling has previously been shown to drive pathogenic neuroinflammation and neuronal cell death in several models of neurological disorders (14, 15, 65–68). Although many studies have reported neuronal necroptosis as a driver of neurodegeneration, we and others have described necroptosis-independent functions for this kinase in the coordination of neuroinflammation (12–17, 69). To date, RIPK3 signaling in astrocytes has received relatively little attention. Our findings here suggest that DAMP signaling activates astrocytic RIPK3 via RAGE signaling, which drives an inflammatory transcriptional program, even in the absence of MLKL. These data suggest that astrocytic RAGE signaling does not induce inflammation via necroptosis, consistent with our prior work showing necroptosis-independent RIPK3 signaling in astrocytes exposed to fibrillar α-synuclein (14).

Future work will be needed to define the signaling events that mediate RAGE-dependent RIPK3 activation. A recent study demonstrated co-immunoprecipitation of RIPK3 with RAGE in an endothelial cell line following stimulation with TNF-α (70), but the nature of this interaction and whether it happens under natural conditions in vivo remains to be established. Although some studies have observed RIPK3 activation downstream of HMGB1 (71, 72), these effects may have been mediated by non-RAGE HMGB1 receptors, such as TLR4, which is known to stimulate RIPK3 via its adaptor molecule, TRIF (73, 74). Both RAGE and RIPK3 signaling appear to converge on the potent activation of NF-κB (33, 75–78), which may provide clues concerning their potential molecular interactions. In any event, delineating the molecular events that promote pathogenic astrocyte activation downstream of DAMP signaling will likely be required to effectively target this pathway for future therapeutic development.

## Methods

### Sex as a biological variable.

For in vivo studies using MPTP, only male mice were used in this study, as female mice exhibit acute toxicity and high rates of mortality following exposure to MPTP (24). Other in vivo studies, including B/B homodimerizer and HMGB1 injection, were performed in balanced groups of both male and female animals. For in vitro studies, primary cells were pooled from both male and female donors or animals. The SH-SY5Y cell line was originally derived from a female donor. Sexually dimorphic phenotypes were not observed in experiments where the sex of experimental subjects was mixed.

### Mouse lines.

Mice were bred and housed under specific pathogen–free conditions in Nelson Biological Laboratories at Rutgers University. *Ripk3*^–/–^ and *Ripk3*^fl/fl^ mouse lines were provided by Genentech, Inc. *Mlkl*^–/–^ (79) and *Ripk3*-2xFV^fl/fl^ (12) lines were provided by Andrew Oberst (University of Washington, Seattle, Washington, USA). *Aldh1l1*-Cre/ERT2 mice were obtained from Jackson Laboratories (Line 031008), and all animals expressing this transgene were treated for 5 days with 60 mg/kg tamoxifen (MilliporeSigma, T5648) in sunflower seed oil (MilliporeSigma, S5007) (i.p.) at least 1 week prior to further experimentation. *Nestin*-Cre mice were obtained from Jackson Laboratories (Line 003771). All genotyping was performed in-house using ear punch tissue lysed overnight in DirectPCR Lysis Reagent (Viagen Biotech, 102-T) and Proteinase K (MilliporeSigma, 3115828001). Sequences for genotyping primers are listed in Supplemental Table 1. PCR bands were visualized on 2% agarose (VWR, 97062) in TBE (VWR, E442) and stained in Diamond Nucleic Acid Stain (Promega, H1181). All experiments were performed in 8- to 12-week-old animals. All transgenic animal lines were backcrossed for at least 10 generations on a C57BL/6J background.

### MPTP model.

MPTP was administered at 20 mg/kg (i.p.) once per day for 5 days (80). Animals were harvested 3 days following the final MPTP injection for gene expression and flow cytometry experiments. Animals were harvested 7 days after the last injection for immunofluorescence detection of neurodegeneration, as well as vertical grid maze studies (Figure 1A). Effective depletion of dopaminergic neurons was assessed via immunostaining for TH, a marker widely used to identify dopamine neurons in models of Parkinson’s disease (24, 81).

### Tissue collection.

Mice were perfused transcardially with ice-cold phosphate-buffered saline (PBS) followed by 4% paraformaldehyde (PFA) for immunofluorescence experiments. Perfused brains were stored in 4% PFA overnight followed by 48 hours in 30% sucrose in PBS. For transcriptional and ELISA studies, mice were perfused with PBS, and midbrain and/or striatal tissues were collected and homogenized for downstream analyses.

### Cell culture and treatment.

Primary human midbrain astrocytes (ScienCell Research Laboratories) were cultured in astrocyte media (ScienCell Research Laboratories, 1801) supplemented with 2% heat-inactivated fetal bovine serum (FBS) (ScienCell Research Laboratories, 0010), astrocyte growth supplement (ScienCell Research Laboratories, 1852), and penicillin/streptomycin (ScienCell Research Laboratories, 0503). Cells from at least 2 distinct donors were used for all experiments. Human neuroblastoma SH-SY5Y cells (ATCC, CRL-2266) were cultured in DMEM (VWR, 0101–0500) supplemented with 10% FBS (Gemini Biosciences, 100–106), nonessential amino acids (Hyclone, SH30138.01), HEPES (Hyclone, 30237.01), penicillin/streptomycin (Gemini Biosciences, 400–110), and amphotericin B antifungal (Gemini Biosciences, 100–104). Differentiation and experimentation occurred in stocks having undergone fewer than 15 passages. SH-SY5Y neuroblastoma cells were differentiated into mature neuron-like cells by treating with retinoic acid (4 μg/mL; MilliporeSigma, R2625) and BDNF (25 ng/mL; MilliporeSigma, B3795) in low-serum (2%) SH-SY5Y media. Differentiated SH-SY5Y cultures were used for experiments 5 to 7 days postdifferentiation. MPP^+^ iodide (MilliporeSigma, D048) was formulated in water to a stock concentration of 500 mM. Recombinant HMGB1 (R&D Systems, 1690-HMB-050) and S100B (human: R&D Systems, 1820-SB; mouse: Novus Biologicals, NBP2-53070) were formulated according to manufacturer recommendations. For cell culture experiments, all recombinant DAMPs were used at a final concentration of 100 ng/mL for 24 hours before collection of preconditioned media and cell lysates. GSK872 was purchased from MilliporeSigma (catalog 530389). FPS-ZM1 was purchased from MilliporeSigma (catalog 55030). JSH-23 was purchased from Selleckchem (S7351). All inhibitors were solubilized in DMSO and used at a final concentration of 1 μM (GSK872 and FPS-ZM1) or 50 μM (JSH-23).

### Primary mouse cell isolation and culture.

Primary mouse midbrain astrocytes were cultured from dissected midbrain tissues derived from mouse pups on postnatal day 3. Tissue was dissociated using Neural Dissociation Kit (T) following manufacturer’s instructions (Miltenyi Biotec, 130-093-231). Midbrain astrocytes were cultured on fibronectin-coated flasks, and nonastrocytic cells were removed via differential adhesion, as previously described (82). Astrocytes were expanded in AM-a medium (ScienCell Research Laboratories, 1831) supplemented with 10% FBS, Astrocyte Growth Supplement-animal (ScienCell Research Laboratories, 1882), and Penicillin/Streptomycin Solution (ScienCell Research Laboratories, 0503). Primary mouse mesencephalic neuron cultures were generated and maintained as described (83, 84). Neurons were cultured for 7 days prior to use in experiments.

### Cell viability test.

Cell viability was assessed with the CellTiter-Glo Luminescent Cell Viability Assay kit (Promega, G7573), according to the manufacturer’s instructions. Luminescence signal was measured with a SpectraMax iD3 plate reader (Molecular Devices).

### Phagocytosis assay.

Differentiated SH-SY5Y neuronal cells were labeled with BioTracker CSFE Cell Proliferation Kit (MilliporeSigma, SCT110) according to the manufacturer’s protocol. Cell death was induced by exposure to TNF-α at 100 ng/mL and cycloheximide (MilliporeSigma, 66-81-9) at 100 μg/mL for 24 hours. Labeled cell debris was collected by centrifugation at 11,000*g* for 10 minutes at 4°C. Unlabeled neuronal debris was used as a staining control. To detect phagocytosis, CSFE-labeled neuronal debris was added to primary midbrain astrocyte cultures at a ratio of 1:100 for 24 hours. Excess neuronal debris was washed away with PBS. Astrocytes were then harvested with cold 5 mM EDTA in PBS followed by scraping of adherent cells. Astrocytes were stained with Zombie NIR (BioLegend) at 1:1,000 in 1× PBS according to the manufacturer’s protocol, followed by fixation in 1% PFA. Phagocytosed CSFE signal was detected using a Northern Lights flow cytometer (Cytek). Analysis was performed by FlowJo software.

### B/B homodimerizer and stereotactic injection.

B/B homodimerizer was purchased from Takara USA (AP20187) and was formulated according to manufacturer’s recommendations. Buprenorphine extended-release (3.25 mg/kg) was administered subcutaneously immediately prior to surgery. Mice were anesthetized with isoflurane (4% induction, 1% maintenance) and positioned on a heating pad while the head was fixed for stereotactic injection. Each animal received 500 nL of freshly formulated B/B homodimerizer or vehicle delivered by a glass pipette using a Programmable Nanoject III Nanoliter Injector (Drummond) unilaterally into the right ventral lateral midbrain (relative to bregma: coordinates anterior/posterior: –3.00 mm, medial/lateral: –1.20 mm, dorsal/ventral: –4.50 mm). The scalp was sutured, and animals were allowed to recover for 24 hours before transcriptional analyses. For in vitro studies, B/B homodimerizer was used at a final concentration of 100 nM.

### qRT-PCR.

Total RNA from homogenized midbrain tissues was extracted using Direct-zol RNA Miniprep kit, following manufacturer’s instructions (Zymo, R2051). Total RNA from cultured cells was isolated using RNeasy Mini Kit according to the manufacturer’s protocol (QIAGEN, 74106). RNA yield and quality of the samples were assessed using a SpectraMax QuickDrop spectrophotometer (Molecular Devices). cDNA was then synthesized with qScript cDNA Synthesis Kit (Quantabio, 95047), followed by qRT-PCR with SYBR Green Master Mix (Bio-Rad, 1725275). Cycle threshold (Ct) values were obtained using a QuantStudio 5 instrument (Applied Biosystems). Delta Ct was calculated as normalized to Ct values of the housekeeping gene 18S (Ct_Target_ − Ct_18S_ = ΔCt). *Z* scores were calculated to graph heatmaps. Primer sequences in our study are listed in Supplemental Table 2.

### Immunofluorescence.

Brains were cryosectioned at 12 mm per slice and mounted on a charged slide. Following thawing in a humidified chamber, tissues were incubated in blocking solution consisting of 5% goat serum (Gibco, 16210) and 0.2% Triton X-100 for 1 hour at room temperature. Sections were then incubated with primary antibody diluted in blocking solution overnight at 4°C in a humidified chamber. Antibodies used in this study are listed in Supplemental Table 3. Slides were then washed 3 times with PBS for 15 minutes followed by incubation in secondary antibody diluted in blocking solution for 1 hour at room temperature. Slides were washed 3 times to remove secondary antibody and were then stained with DAPI (Biotium, 40011) diluted in PBS for 20 minutes at room temperature, followed by another wash. Sections were coverslipped with Prolong Diamond Antifade Mountant medium (Invitrogen, P36930). Slides were allowed to dry and images were acquired using Airyscan fluorescent confocal microscope (Carl Zeiss, LSM 800).

### Flow cytometry.

After perfusing with ice-cold PBS, mouse midbrains were dissected and minced with a blade. Tissues were then further homogenized via 30-minute incubation in prewarmed digestion buffer consisting of 2% FBS, 1% glutamine, 1% nonessential amino acids, 1% penicillin/streptomycin/amphotericin, and 1.5% HEPES, with 0.7 U/mL collagenase VIII and 50 U/mL DNase I on an orbital shaker. Triturated tissue homogenate was then passed through a 70 µm cell strainer (VWR, 76327-100) and centrifuged at 350*g* at 4°C for 10 minutes to obtain a single-cell suspension. Cell gradient separation was then achieved by resuspending the pellet in 20% bovine serum albumin (BSA) in DMEM followed by 20-minute centrifugation at 1,200*g* at 4°C. After removing the myelin layer, the cell gradient was disrupted by inverting in additional FACS buffer that consisted of 1 mM EDTA in PBS with 1% BSA. Resuspended cells were then incubated in antibodies for 30 minutes at 4°C in the dark. Antibodies used in this study are listed in Supplemental Table 3. After washing with cold FACS buffer, cold 1% PFA was used to fix the cells. Data collection and analysis were performed using a Cytek Northern Lights Cytometer and FlowJo software. Data were normalized using standard counting beads (Thermo Fisher Scientific, C36950).

### ELISA.

The following ELISA kits were used according to the manufacturer’s instructions: HMGB1 (Novus Biologicals, NBP2-62766), Phospho-MLKL (RayBiotech, PEL-MLKL-S345-1), and Phospho-NFκB p65 (Thermo Fisher Scientific, 85-86082-11).

### FLAG pulldown and Western blot.

Pulldown of FLAG-tagged RIPK3-2xFV protein was performed using a DYKDDDDK Isolation Kit (Miltenyi Biotec, 130-101-591) according to manufacturer’s instructions. DSS cross-linking was performed as described (35) using DSS cross-linking reagent (Thermo Fisher Scientific, A39267). Western blot was performed as described (85) using antibodies against RIPK3 (Cell Signaling Technology, 957025), MLKL (MilliporeSigma, MABC604), and Actin (MilliporeSigma, SAB3500350).

### LC-MS.

A single dose of MPTP (40 mg/kg) was administered for liquid chromatography-mass spectrometry (LC-MS) analysis of MPP^+^ in vivo. Mice were transcardially perfused with ice-cold PBS 90 minutes after MPTP injection. Whole brain tissues were then isolated and homogenized in CryoMill tubes containing cold 40:40:20 methanol/acetonitrile/water solution with 0.5% formic acid. Following a 10-minute incubation on ice, tissue homogenates were centrifuged in the cold room for 10 minutes at 16,000*g*. Supernatants were then transferred to a new collection tube. The final sample was then treated with 15% NH_4_HCO_3._ LC-MS was performed at the Metabolomics Shared Resource Core Facility at the Rutgers Cancer Institute of New Jersey.

### Behavioral assessment.

The vertical grid motor assessment task was adapted from previous work (29). Briefly, mice were acclimated to the vertical grid apparatus 3 times a day for 2 consecutive days. On each day, each mouse was placed on the inside of the apparatus 3 cm from the top, facing upward, and was allowed to turn around and climb down. The trial was repeated whenever the mouse failed to climb down or turn around within 60 seconds. The same trials were repeated on the day following acclimation and video-recorded for analysis.

### Bulk RNA-Seq.

Total RNA from midbrain tissues was extracted and assessed as described above. RNA samples were sent to Azenta for library preparation and next-generation sequencing. RNA yield and sample quality were assessed with Qubit (Invitrogen) and TapeStation (Agilent). The Illumina HiSeq platform and 2 × 150 bp paired-end reads were used for the RNA-Seq. Initial analysis was processed by Azenta. The quality of raw RNA-Seq data (FASTQ) files were evaluated using FASTQC. Sequence reads were trimmed to remove possible adapter sequences and nucleotides with poor quality using Trimmomatic v.0.36. Trimmed reads were then mapped to the mouse reference genome (GRCm38) available on ENSEMBL using the STAR aligner v.2.5.2b. Unique gene hit counts were calculated by using featureCounts from the Subread package v.1.5.2. The gene hit counts table was used for downstream differential expression analysis via DESeq2. Further statistical analysis was performed using R.

### Image analysis.

To quantify TH^+^ and SMI32^+^ puncta and colocalization, images were processed by Imaris software (Oxford Instruments, Bitplane 9.5). Object-based colocalization was used with the “Coloc” feature. For TH^+^ and SMI32^+^ particles, the spot detection function was used to define particles by first creating “vesicles” in each channel. Input intensity for threshold was chosen to best represent the signal for both channels. Colocalized particles were defined with the “classification” feature, where the distance between TH^+^ and SMI32^+^ particles within 1 μm or less is considered colocalization. The percentage area and mean intensity of GFAP^+^ and IBA1^+^ signal were assessed using Fiji (ImageJ; NIH) software.

### Statistics.

Statistical analysis was completed using GraphPad Prism 9. Normally distributed data were analyzed using appropriate parametric tests: Student’s *t* test (2 tailed) or 2-way ANOVA with Tukey’s multiple comparisons test were used to determine significant differences between groups. A *P* value less than 0.05 was considered statistically significant. All data points represent biological replicates unless otherwise noted.

### Study approval.

All animal experiments were performed with approval of the Rutgers University Institutional Animal Care and Use Committee.

### Data availability.

Numerical data associated with this study can be found in the Supporting Data Values file. RNA-Seq data generated in this study are deposited in NCBI’s Gene Expression Omnibus and can be accessed under accession number GSE237891.

## Author contributions

NPC and BPD conceived the study; NPC, EMD, ML, IE, TWC, WRE, MN, MM, DA, CA, and BPD investigated; NPC, EMD, ML, IE, TWC, MM, and BPD analyzed data; AWK, RH, and BPD provided resources; NPC and BPD wrote the original draft; NPC, EMD, TWC, CA, and BPD reviewed and edited the manuscript; CA, AWK, RH, and BPD supervised; and RH and BPD acquired funding.

## Supplementary Material

Supplemental data

Unedited blot and gel images

Supporting data values

## Version 1 05/07/2024

In-Press Preview

## Version 2 06/10/2024

Electronic publication
