# Supplementary material for: Neuronal DAMPs exacerbate neurodegeneration via astrocytic RIPK3 signaling
Source: JCI Insight. 2024 May 7;9(11):e177002. doi: 10.1172/jci.insight.177002 (PMC11382884; doi:10.1172/jci.insight.177002)
Supplement: Supplemental data [file jciinsight-9-177002-s084.pdf]

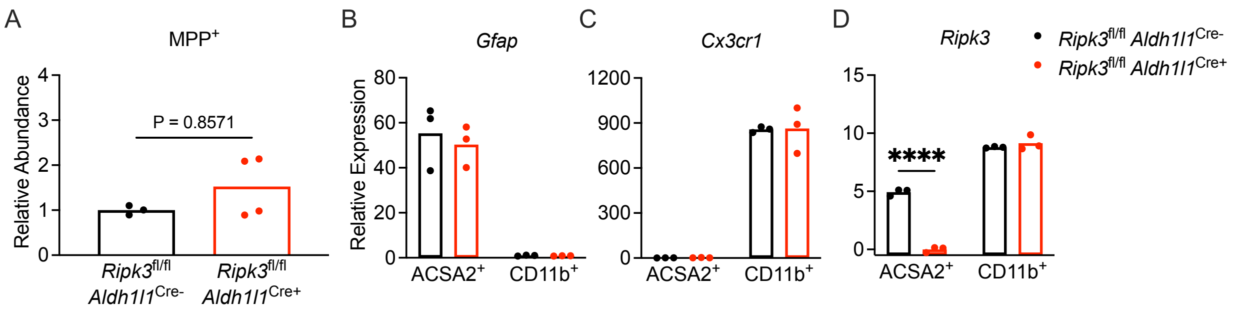


**Supplemental Figure 1.** ***Deletion of astrocytic Ripk3 does not impact MPTP metabolism in vivo.*** **(A)** LC-MS analysis of $MPP^+$ abundance in midbrain homogenates of mice of indicated genotypes 90 minutes following intraperitoneal MPTP injection. (B-C) ACSA2+ (astrocytes) and CD11b+ (microglia) cells were isolated from brains of mice of indicated genotypes via magnetic activated cell sorting (MACS). Purity was confirmed via qRT-PCR detection of cell type specific genes (*Gfap* for astrocytes; *Cx3cr1* for microglia) **(B-C)**. **(D)** qRT-PCR detection of *Ripk3* in sorted cell populations. ****p<0.0001.

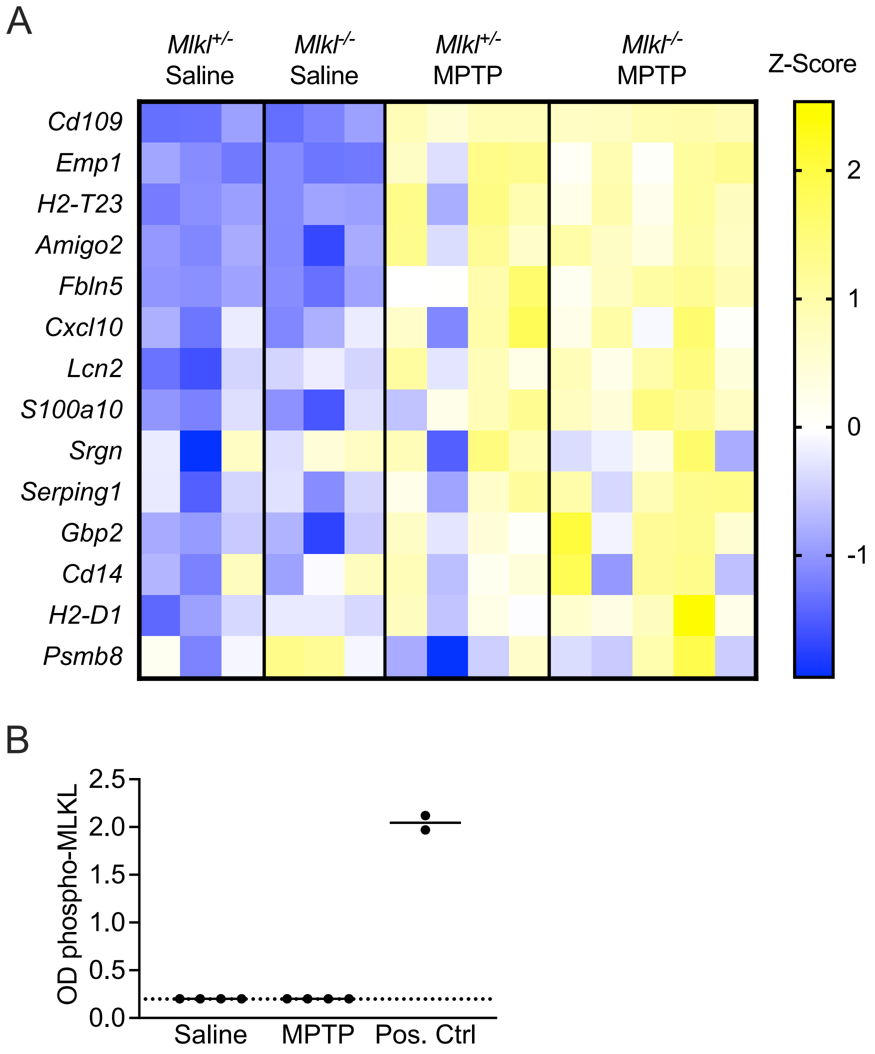


**Supplemental Figure 2.** ***Genes associated with astrocyte activation are not impacted by loss of MLKL in the MPTP model.*** A) qRT-PCR analysis of indicated genes in midbrain homogenates of *Mlkl* knockout and heterozygous littermate control animals 3 days post treatment with MPTP or saline. B) ELISA detection of phospho-MLKL (ser-345) in midbrain homogenates of WT mice 3 days post treatment with MPTP or saline. Positive control represents calyculin-treated HT29 cell lysate provided in kit.

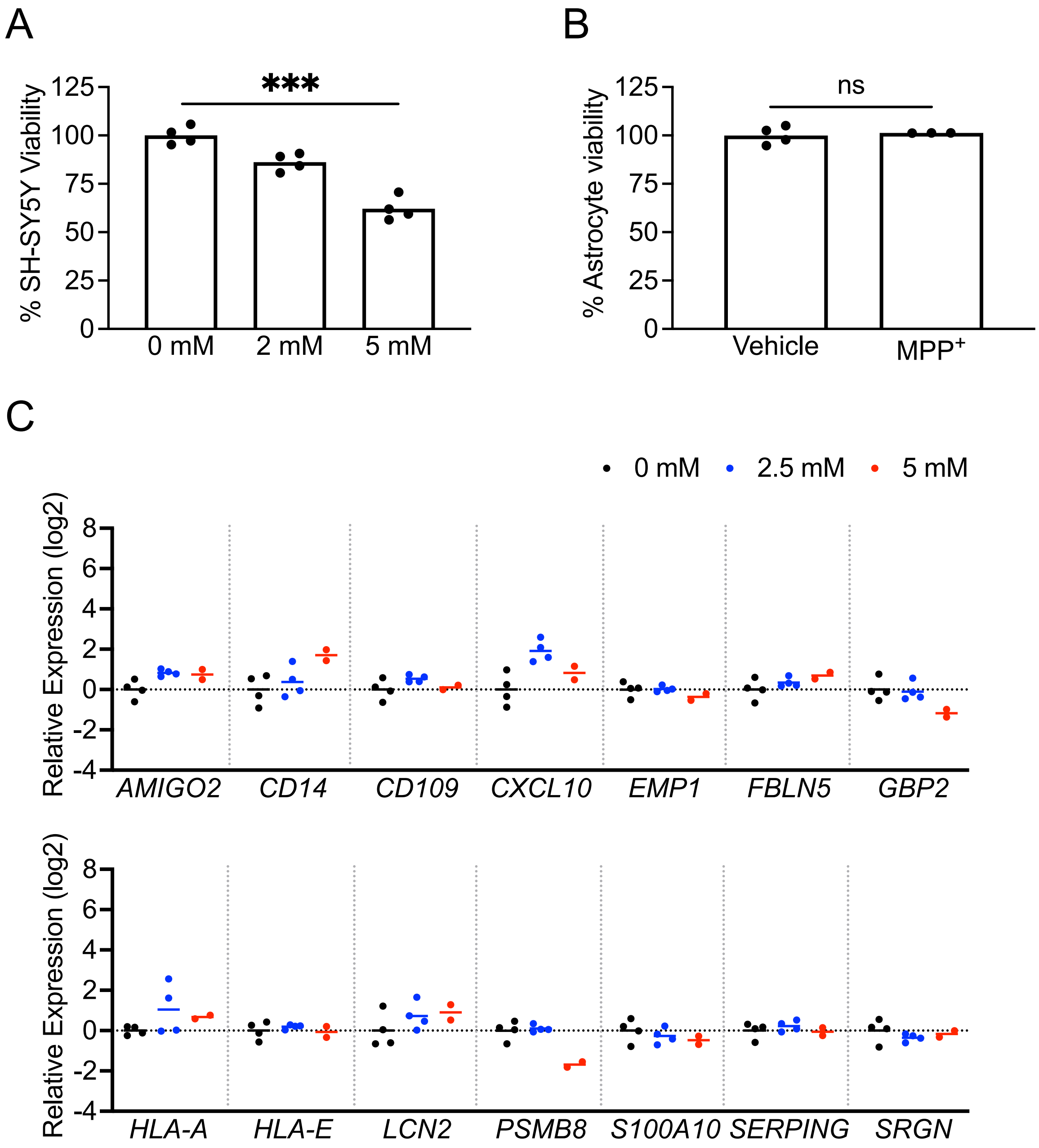


**Supplemental Figure 3.** ***MPP⁺ induces death in SH-SY5Y cells, but does not induce death or transcriptional activation in astrocytes.*** **(A-B)** Cell Titer Glo analysis of viability in SH-SY5Y **(A)** or primary human midbrain astrocyte **(B)** cultures treated with 2 or 5 mM **(A)** or 2.5 mM **(B)** $MPP^+$ for 24h. **(C)** qRT-PCR analysis of indicated genes in primary human midbrain astrocytes treated with 2.5 mM $MPP^+$ for 24h. *** $p<0.001$.

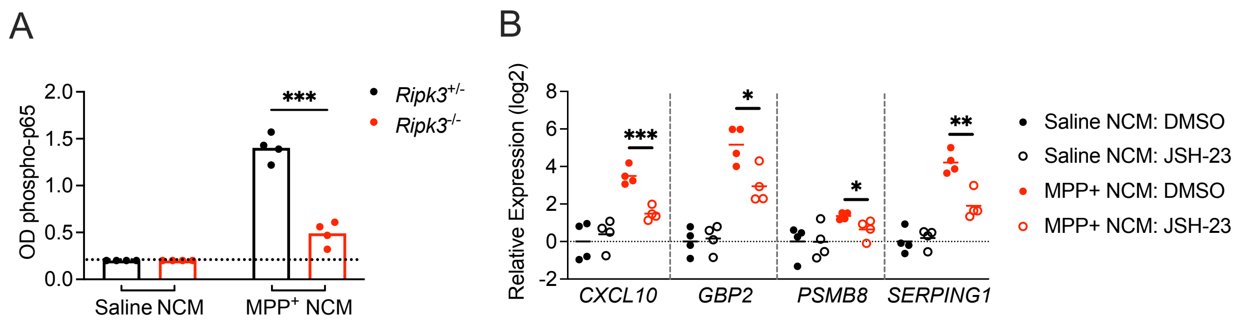


**Supplemental Figure 4.** ***MPP$^+$* NCM induces RIPK3-dependent NFkB activation and NFkB-mediated gene expression.** A) Primary human midbrain astrocytes of indicated genotypes were treated with NCM and phospho-p65 was detected via ELISA 4h post treatment. B) qRT-PCR analysis of indicated genes in primary human midbrain astrocytes treated for 24h with indicated conditions. *$p<0.05$, **$p < 0.01$, ***$p < 0.001$.

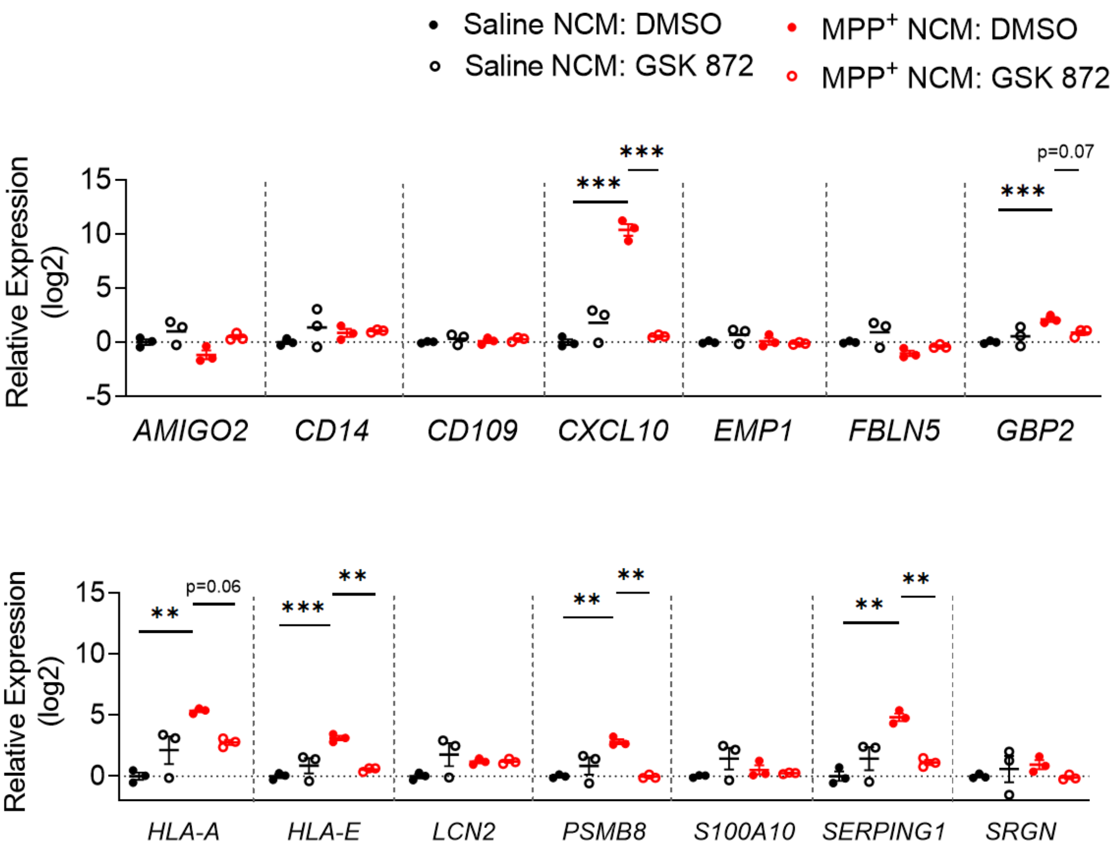


**Supplemental Figure 5.** ***Astrocytes maintain transcriptional activation for at least 24 hours following removal of MPP⁺ NCM.*** qRT-PCR analysis of indicated genes in primary human midbrain astrocytes treated for 24h with indicated conditions, followed by washing, addition of fresh culture medium (with no treatment), and an additional 24h incubation before harvest. *p<0.05, **p < 0.01, ***p < 0.001.

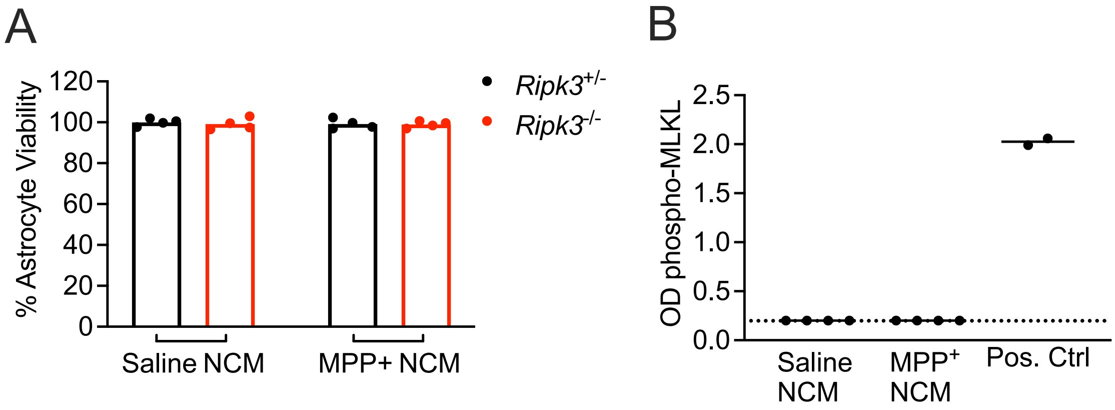


**Supplemental Figure 6.** ***MPP$^+$ NCM does not induce cell death or phosphorylation of MLKL in primary midbrain astrocytes.*** A) Primary murine midbrain astrocytes of indicated genotypes were treated with NCM and viability was assessed after 24h via Cell Titer Glo. B) ELISA detection of phospho-MLKL (ser-345) in midbrain astrocyte cultures 24h post treatment with NCM. Positive control represents calyculin-treated HT29 cell lysate provided in kit.

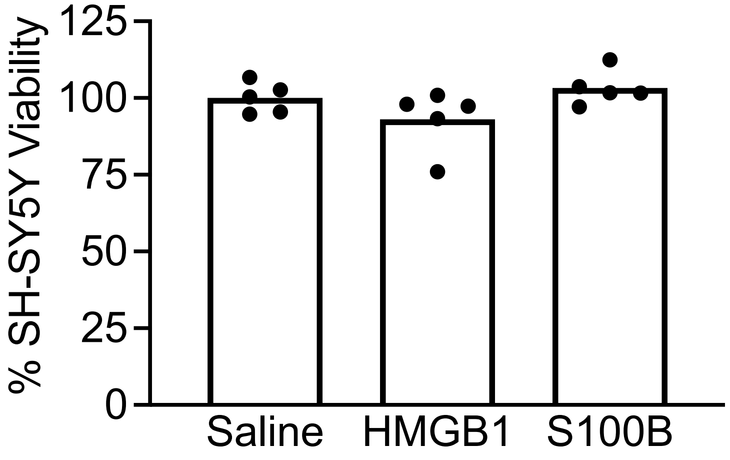


**Supplemental Figure 7.** ***Recombinant DAMPs are not intrinsically toxic to SH-SY5Y cells.*** **(A-B)** Cell Titer Glo analysis of viability in SH-SY5Y treated with indicated DAMP ligand for 24 hours.

## Supplemental Table 1.

### Primer sequences for genotyping

| Target | Note | Primer Sequence (5'-3') | Product size |
|---|---|---|---|
| *Aldh1l1*-Cre/ERT2 | Internal control Forward | CTGTCCCTGTATGCCTCTGG | 415bp |
| | Internal control reverse | AGATGGAGAAAGGACTAGGCTACA | |
| | Transgene Forward | CTTCAACAGGTGCCTTCCA | 198bp |
| | Transgene Reverse | GGCAAACGGACAGAAGCA | |
| *Mlkl*$^{-/-}$ | MLKL_001 | TATGACCATGGCAACTCACG | WT 498bp<br>KO 158bp |
| | MLKL_002 | ACCATCTCCCCAAACTGTGA | |
| | MLKL_003 | TCCTTCCAGCACCTCGTAAT | |
| *Nestin*-Cre | WT Forward | TTGCTAAAGCGCTACATAGGA | WT 246bp<br>Cre 150bp |
| | Common Reverse | GCCTTATTGTGGAAGGACTG | |
| | Transgene Forward | CCTTCCTGAAGCAGTAGAGCA | |
| *Ripk3*$^{-/-}$ | RIP3_001 | CGCTTTAGAAGCCTTCAGGTTGAC | WT 733bp<br>KO 485bp |
| | RIP3_002 | GCAGGCTCTGGTGACAAGATTCATGG | |
| | RIP3_003 | CCAGAGGCCACTTGTGTAGCG | |
| *Ripk3*$^{fl/fl}$ | R3FL_001 | ACGATGTCTTCTGTCAAGTTATG | WT 300bp<br>LoxP 334bp |
| | R3FL_002 | CAGTTCTTCACGGCTCAC | |
| | R3FL_003 | TCTGGTAAGGAGGGTCAC | |
| *Ripk3*-2xFV$^{fl/fl}$ | ROSA Forward | AGCACTTGCTCTCCCAAAGTC | 346bp |
| | ROSA Reverse | CCGACAAAACCGAAAATCTGTGGG | |
| | Transgene Forward | CGCTTTAGAAGCCTTCAGGTTGAC | 349bp |
| | Transgene Reverse | GCAGGCTCTGGTGACAAGATTCATGG | |

**Supplemental Table 2.**

**Primer sequences for qRT-PCR studies**

| Target | Forward | Reverse |
|---|---|---|
| *18S* hu | AGAAACGGCTACCACATCCA | CCCTCCAATGGATCCTCGTT |
| *18s* ms | CTTAGAGGGACAAGTGGCG | ACGCTGAGCCAGTCAGTGTA |
| *AMIGO2* hu | CTTCAGCGTTTGGAGGGCT | CAGGGAACAGTCACAGACAAAT |
| *Amigo2* ms | GAGGCGACCATAATGTCGTT | GCATCCAACAGTCCGATTCT |
| *CCL2* hu | GCAGCAAGTGTCCCAAAGAA | CTGGGGAAAGCTAGGGGAAA |
| *CD109* hu | CAGGAATGTGGACTCTGGGT | CTTTCGGACATGTGGACTGC |
| *CD109* ms | CACAGTCGGGAGCCCTAAAG | GCAGCGATTTCGATGTCCAC |
| *CD14* hu | CCGCTGTGTAGGAAAGAAGC | GCAGCGGAAATCTTCATCGT |
| *CD14* ms | GGACTGATCTCAGCCCTCTG | GCTTCAGCCCAGTGAAAGAC |
| *CXCL10* hu | GTGGCATTCAAGGAGTACCTC | TGATGGCCTTCGATTCTGGATT |
| *Cxcl10* ms | CCCACGTGTTGAGATCATTG | CACTGGGTAAAGGGGAGTGA |
| *EMP1* hu | CCAGTACACCAGCAGAGGAA | AACAGTAGCGATGTGGACCA |
| *Emp1* ms | GAGACACTGGCCAGAAAAGC | TAAAAGGCAAGGGAATGCAC |
| *FBLN5* hu | TCGCCAGTCAGGACAGTGT | AGTAGGGGTTCGAGTAGGGC |
| *Fbln5* ms | CTTCAGATGCAAGCAACAA | AGGCAGTGTCAGAGGCCTTA |
| *GBP2* hu | CTATCTGCAATTACGCAGCCT | TGTTCTGGCTTCTTGGGATGA |
| *Gbp2* ms | GGGGTCACTGTCTGACCACT | GGGAAACCTGGGATGAGATT |
| *HLA-A* hu | GACCAGGAGACACGGAATGTG | CCTCGTTCAAGGCGATGTAATC |
| *HLA-E* hu | TTCCGAGTGAATCTGCGGAC | GTCGTAGGCGAACTGTTCATAC |
| *H2-D1* ms | TCCGAGATTGTAAAGCGTGAAGA | ACAGGGCAGTGCAGGGATAG |
| *H2-T23* ms | GGACCGCGAATGACATAGC | GCACCTCAGGGTGACTTCAT |
| *LCN2* hu | GAAGTGTGACTACTGGATCAGGA | ACCACTCGGACGAGGTAACT |
| *Lcn2* ms | CCAGTTCGCCATGGTATTTT | CACACTCACCACCCATTCAG |
| *PSMB8* hu | GGTCCTACATTAGTGCCTTACGG | CGCAGATAGTACAGCCTGCATT |
| *Psmb8* ms | CAGTCCTGAAGAGGCCTACG | CACTTTCACCCAACCGTCTT |
| *S100A10* hu | ATGAAGGACCTGGACCAGTG | GCAGATTCCTTAAGCGACCC |
| *S100a10* ms | CCTCTGGCTGTGGACAAAAT | CTGCTCACAAGAAGCAGTGG |
| *SERPING1* hu | GGGATGCTTTGGTAGATTTCTCC | GAGGATGCTCTCCAGGTTTGT |
| *Serping1* ms | ACAGCCCCCTCTGAATTCTT | GGATGCTCTCCAAGTTGCTC |
| *SRGN* hu | GGACTACTCTGGATCAGGCTT | CAAGAGACCTAAGGTTGTCATGG |
| *Srgn* ms | GCAAGGTTATCCTGCTCGGA | TGGGAGGGCCGATGTTATTG |

## Supplemental Table 3.

| | | |
|---|---|---|
| Flow cytometry antibodies | | |
| CD11b | BioLegend | Clone M1/70 |
| CD45.2 | BioLegend | Clone 104 |
| CD80 | BioLegend | Clone 16-10A1 |
| F4/80 | BioLegend | Clone BM8 |
| MHC-II | BioLegend | Clone M5/114.15.2 |
| Zombie NIR | BioLegend | #423105 |
| BioTracker CSFE | Sigma-Aldrich | SCT110 |
| Immunofluorescence antibodies | | |
| Rat anti-GFAP | Invitrogen | 13-0300 |
| Rabbit anti-IBA-1 | Wako Chemicals | 1919741 |
| Chicken anti-tyrosine hydroxylase | Aves Labs | TYH |
| Rabbit anti-tyrosine hydroxylase | Abcam | Ab112 |
| Mouse anti-SMI32 | BioLegend | 801701 |
| Chemicals, peptides, and recombinant proteins | | |
| Human BDNF | Sigma-Aldrich | B3795 |
| GSK872 | Millipore Sigma | 530389 |
| FPS-ZM1 | Sigma-Aldrich | 55030 |
| Retinoic acid | Sigma-Aldrich | R2625 |
| Cyclohexamide | Sigma-Aldrich | 66-81-9 |
| Recombinant HMGB1 | R&D Systems | 1690-HMB-050 |
| Recombinant mouse S100β | Novus Biologicals | NBP2-53070 |
| B/B Homodimerizer | Takara USA Inc. | AP20187 |
| MPP+ iodide | Sigma-Aldrich | D048-100MG |
| JSH-23 | Selleck Chem | S7351 |
| Anti-HMGB1 nAb | Arigobio | ARG66714 |
