# Supplementary material for: Neuronal DAMPs exacerbate neurodegeneration via astrocytic RIPK3 signaling
Source: JCI Insight. 2024 May 7;9(11):e177002. doi: 10.1172/jci.insight.177002 (PMC11382884; doi:10.1172/jci.insight.177002)

Figure 6E

1	2	3	4	5	6	7	8	9
Ladder	Saline NCM 1	Saline NCM 1 DSS	Saline NCM 2	Saline NCM 2 DSS	NCM MPP+ 1	NCM MPP+ 1 DSS	NCM MPP+ 2	NCM MPP+ 2 DSS

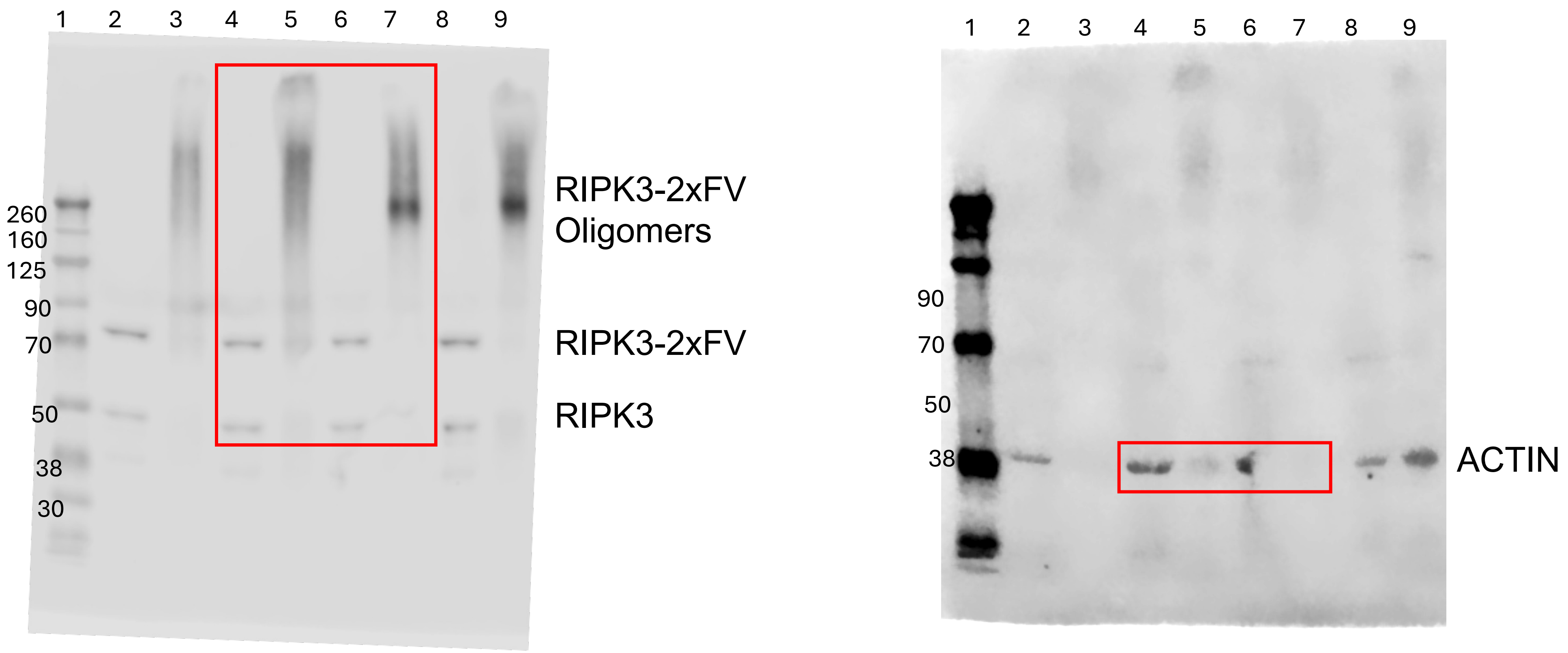

Figure 6F

1	2	3	4	5	6	7	8	9	10	11	12	13
Ladder	Saline NCM 1 Input	Saline NCM 1 Flowthrough	Saline NCM 1 Eluate	Saline NCM 2 Input	Saline NCM 2 Flowthrough	Saline NCM 2 Eluate	NCM MPP+1 Input	NCM MPP+1 Flowthrough	NCM MPP+1 Eluate	NCM MPP+2 Input	NCM MPP+2 Flowthrough	NCM MPP+2 Eluate

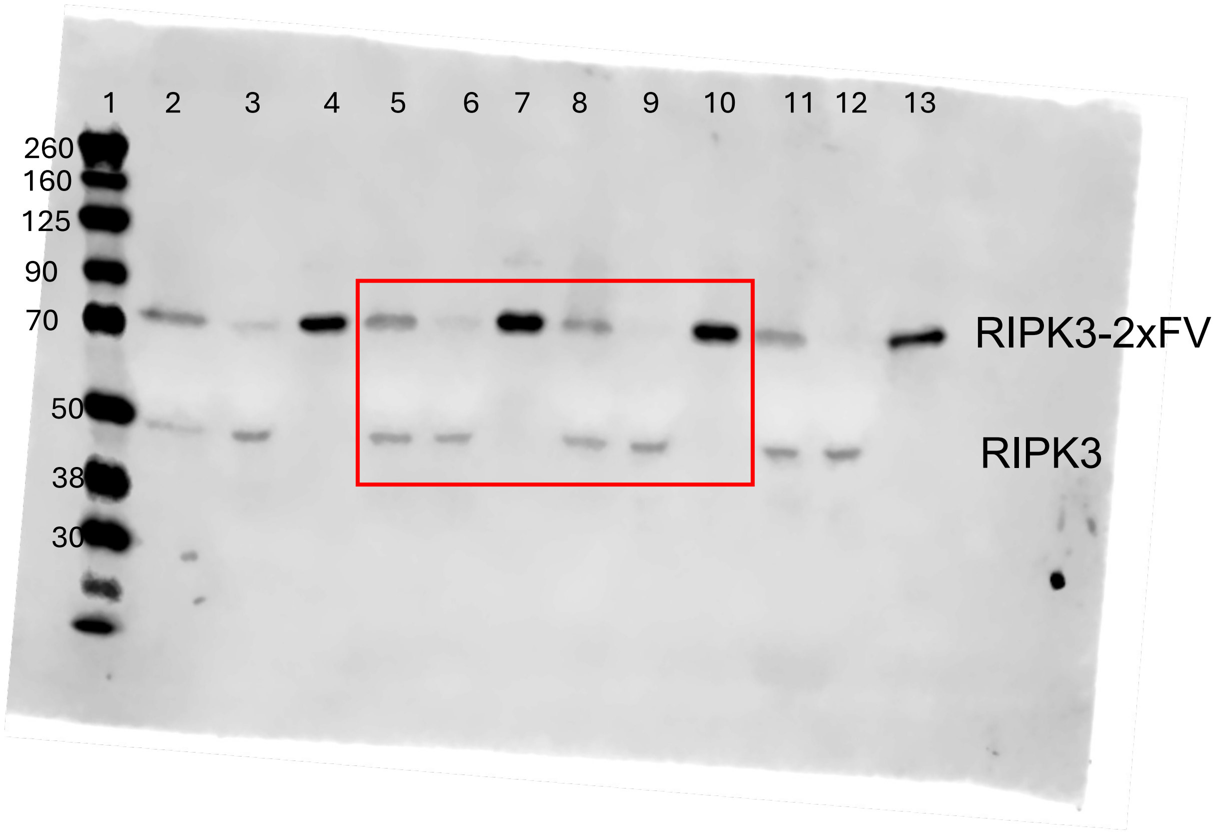

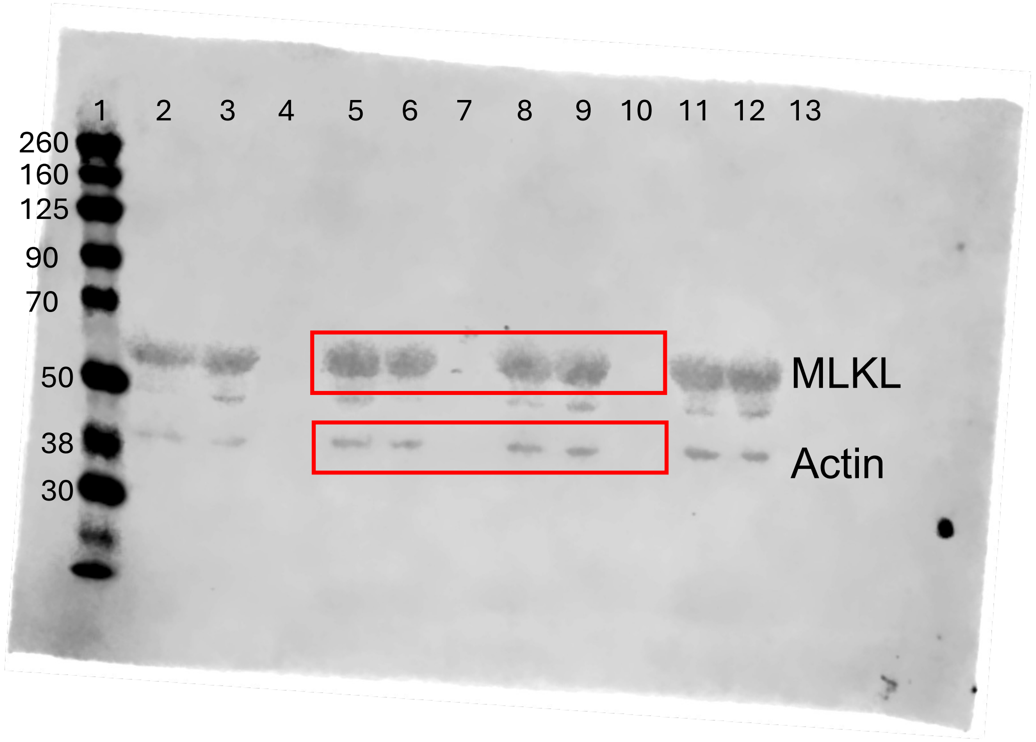

Supplement: Unedited blot and gel images [file jciinsight-9-177002-s085.pdf]
